# Studies of OC-STAMP in Osteoclast Fusion: A New Knockout Mouse Model, Rescue of Cell Fusion, and Transmembrane Topology

**DOI:** 10.1371/journal.pone.0128275

**Published:** 2015-06-04

**Authors:** Hanna Witwicka, Sung-Yong Hwang, Pablo Reyes-Gutierrez, Hong Jia, Paul E. Odgren, Leah Rae Donahue, Mark J. Birnbaum, Paul R. Odgren

**Affiliations:** 1 Department of Cell and Developmental Biology, University of Massachusetts Medical School, Worcester, MA, United States of America; 2 Parallax Pictures, Princeton, MA, United States of America; 3 The Jackson Laboratory, Bar Harbor, ME, United States of America; 4 Department of Biology, Merrimack College, North Andover, MA, United States of America; Charles P. Darby Children's Research Institute, UNITED STATES

## Abstract

The fusion of monocyte/macrophage lineage cells into fully active, multinucleated, bone resorbing osteoclasts is a complex cell biological phenomenon that utilizes specialized proteins. OC-STAMP, a multi-pass transmembrane protein, has been shown to be required for pre-osteoclast fusion and for optimal bone resorption activity. A previously reported knockout mouse model had only mononuclear osteoclasts with markedly reduced resorption activity in vitro, but with paradoxically normal skeletal micro-CT parameters. To further explore this and related questions, we used mouse ES cells carrying a gene trap allele to generate a second OC-STAMP null mouse strain. Bone histology showed overall normal bone form with large numbers of TRAP-positive, mononuclear osteoclasts. Micro-CT parameters were not significantly different between knockout and wild type mice at 2 or 6 weeks old. At 6 weeks, metaphyseal TRAP-positive areas were lower and mean size of the areas were smaller in knockout femora, but bone turnover markers in serum were normal. Bone marrow mononuclear cells became TRAP-positive when cultured with CSF-1 and RANKL, but they did not fuse. Expression levels of other osteoclast markers, such as cathepsin K, carbonic anhydrase II, and NFATc1, were not significantly different compared to wild type. Actin rings were present, but small, and pit assays showed a 3.5-fold decrease in area resorbed. Restoring OC-STAMP in knockout cells by lentiviral transduction rescued fusion and resorption. N- and C-termini of OC-STAMP were intracellular, and a predicted glycosylation site was shown to be utilized and to lie on an extracellular loop. The site is conserved in all terrestrial vertebrates and appears to be required for protein stability, but not for fusion. Based on this and other results, we present a topological model of OC-STAMP as a 6-transmembrane domain protein. We also contrast the osteoclast-specific roles of OC- and DC-STAMP with more generalized cell fusion mechanisms.

## Introduction

Bone resorbing osteoclasts are unusual, but not unique, in that they are polykaryons formed by the fusion of mononuclear precursor cells. Hematopoietic cells of the monocyte-macrophage lineage can give rise by fusion to either osteoclasts or foreign body giant cells (FBGC), depending on extracellular signals. The regulation of cell-cell fusion is of particular interest in osteoclasts since their functions of bone resorption and secretion of digested bone are so dependent upon extremely active membrane dynamics, including formation of the ruffled border and high rates of endocytosis, vesicle fusion, and transcytosis [1–5]. In recent years, more detailed and deeper insights into osteoclast fusion have emerged through studies of specific fusion factors in vivo and in vitro, and through dissecting both the timing and the molecular and cellular steps involved. A first step is the recruitment of mononuclear precursors to specific sites via RANKL-induced expression of chemokines and receptors by pre-osteoclasts [6]. Next, a step-wise process has been described in several reports by Søe and co-workers which entails the action of syncytin1, CD47, and DC-STAMP in early fusion events, and of connexin 43 at a later stage in the engulfment of mononuclear cells by more mature, multinucleated osteoclasts [7,8]. Those authors suggest that cells choose fusion partners which are in different, heterogeneous states of differentiation. Other factors have also been shown to be essential. A recent study identified a novel role for the intracellular adapter protein, Tks5, in cell fusion in osteoclasts and cancer cells, acting downstream of PI3-kinase and Src to mediate formation of circumferential podosomes and localization of specific phosphoinositides to the fusing domains of the plasma membrane [9]. Another recent report found that dynamin and endocytotic processes were required for fusion of both pre-osteoclasts and myoblasts [10].

Besides these factors, which are mostly ubiquitously, or at least widely, expressed, two related transmembrane proteins, which are known to be essential for pre-osteoclast fusion, are restricted to pre-osteoclasts and pre-foreign body giant cells (FBGC): dendritic cell-specific transmembrane protein (DC-STAMP) [11–13] and osteoclast-stimulatory transmembrane protein (OC-STAMP) [14,15]. Neither protein has homology to the other fusion factors described above. The “STAMPs” are both very strongly induced during stimulation of osteoclast differentiation by RANKL or FBGC by GM-CSF [13,15,16], and their expression has only been detected in monocyte/macrophage lineage cells. Both are predicted to be multiple-pass transmembrane proteins with little direct amino acid homology to each other, but with strong similarity in predicted secondary structure [15]. Transmembrane topology prediction algorithms yield several models for intra- and extracellular orientation and for the number of transmembrane domains for both OC- and DC-STAMP. Although some analyses have predicted a 7-pass transmembrane structure for DC-STAMP [17], the most frequent prediction for DC- and OC-STAMP is 6 transmembrane domains with both the N- and C-termini residing in the cytoplasm (e.g., see [15]).

Interestingly, studies of cells from homozygous knockout (KO) mice found that each of the STAMPs is required on only one cell undergoing fusion [13,14], showing that they cannot be forming “fusion bridges” to themselves across the cell-cell junction. Mononuclear osteoclasts from each knockout strain were shown in pit forming assays to be highly deficient in bone resorption capacity. *Dcstamp* ^-/-^ cells resorbed about 3-fold less area than wild-type (WT) cells, and *Ocstamp* ^*-/-*^ cells resorbed about 6-fold less [13,14]. Consistent with this, the DC-STAMP KO mice also had a roughly 3-fold increase in trabecular bone in the metaphysis compared to WT animals. Unexpectedly, however, the OC-STAMP KO mice were reported to have no changes in skeletal parameters despite the loss of pit-forming ability. A few individual animals appeared to have increased trabecular bone in the femoral metaphysis, but not enough for statistically significant changes [14]. That report, however, did not provide age, gender, or cohort size information on the mice analyzed.

BLAST searches identify OC-STAMP genes in all mammals, amphibians, reptiles, and birds for which sequence data are available. Interestingly, these presumed orthologs all carry a conserved putative glycosylation site in what is predicted to be an extracellular loop by most transmembrane analytical algorithms [15]. Mechanistic understanding of OC-STAMP and DC-STAMP will require clearer knowledge of their membrane topology and of potential functions of post-translational modifications. This in turn will provide insights into their roles in bone turnover and skeletal maintenance in vivo. To address these and other related questions, we undertook making an additional knockout mouse line. Here we describe its skeletal and osteoclast phenotype, and we present investigations of OC-STAMP function, topology, and post-translational modifications.

## Materials and Methods

### Animals

All animals were obtained from our colonies of C57BL/6J mice maintained at the University of Massachusetts Medical School under specific-pathogen-free conditions, and all procedures were in accordance with the NIH Guide for the Care and Use of Laboratory animals and were approved by the Institutional Animal Care and Use Committee of the University of Massachusetts Medical School. Euthanasia was performed by inhalation anesthesia followed by decapitation.

### Gene targeting

**Fig 1:**
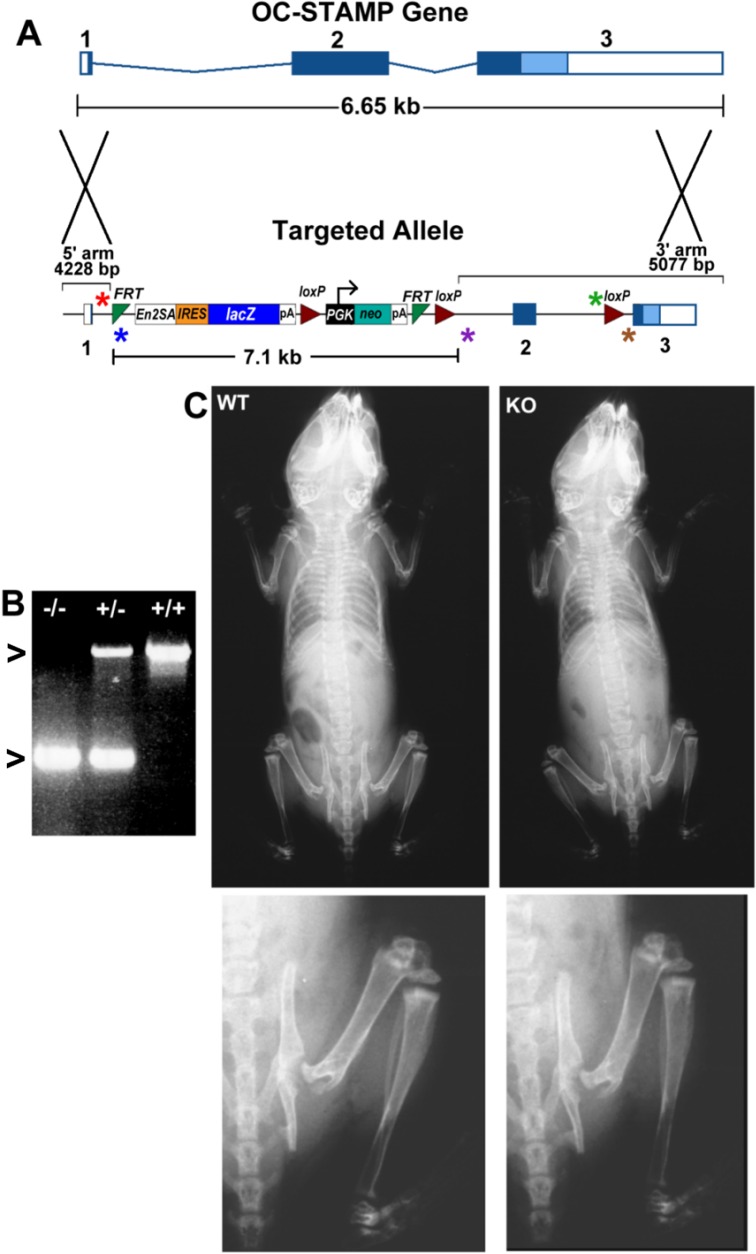
OC-STAMP knockout allele, radiographs. **A.** Gene targeting vector is a “knockout first” homologous recombination gene trap with a selection cassette and recombination sites for Cre (loxP) and flippase (Frt), as indicated. This allele, which results in a complete loss-of-function, is designated *Ocstamp* ^*tm1a(KOMP)Wtsi*^. Exons in the upper (gene) and lower (targeting construct) diagrams are numbered 1, 2, and 3. The selection cassette in intron 1 eliminates production of any functional OC-STAMP. The 5’ and 3’ UTR’s are shown in boxes, and the 3’ alternative end is shown in light blue. Asterisks indicate locations of genotyping primers. From left to right they are: OC-F1 (red) (5’-TTGCCTGTAAATGATGGAGTGGGC-3’); En2R1 (blue) (5’-TGGTGTGGGAAAGGGTTCGAAGTT-3’); OCR1E (purple) (5’-TGGCGCAGCTGGTAAGTGGTATTA-3’). These give PCR products of 1044 bp from WT (OCF1 and OCR1E) and 297 bp from the knockout allele (OCF1 and En2R1). To confirm correct 3’ end insertion, the right-hand pair of primers are: LoxPF (green) (5’-GAGATGGCGCAACGCAATTAAT-3’) and SR1 (brown) (5’-CTGTGACTAAGTAACCATCAAAGCGG-3’), which give a 681 bp product only in the targeted allele (not shown). **B.** PCR of genomic DNA from the mice yielded the expected sizes in homozygous wild type (+/+; upper arrowhead indicates 1044 bp), heterozygotes (+/-), and homozygous knockout (-/-; lower arrowhead indicates 297 bp). **C.** X-rays of 2-week-old wild type (left) and OCSt-KO mice showed no differences in either whole-body views (upper) or rear quadrant views (lower panels). All skeletal elements appear normally formed and without accumulation of trabecular or cortical bone.

We obtained targeted mouse ES cells from the Knockout Mouse Project (KOMP) consortium. The “knockout first” gene trap allele used standard homologous recombination and is shown in **Fig 1**. Blastocyst injection yielded several chimeras, and we had germline transmission in two of them. The mice have been bred and maintained in the C57BL/6J strain. The KOMP nomenclature convention for the targeted allele shown in **Fig 1** is: *Ocstamp* ^*tm1a(KOMP)Wtsi*^. Genotyping was done by PCR using the following primers:

OC-F1 (5’-TTGCCTGTAAATGATGGAGTGGGC-3’); En2R1 (5’-TGGTGTGGGAAAGGGTTCGAAGTT-3’); OCR1E (5-TGGCGCAGCTGGTAAGTGGTATTA-3’). These gave PCR products of 1044 bp from WT (OCF1 and OCR1E) and 297 bp from the knockout allele (OCF1 and En2R1). To confirm correct 3’ end insertion of the gene trap, the right-hand pair of primers were: LoxPF (5’-GAGATGGCGCAACGCAATTAAT-3’) and SR1 (5’-CTGTGACTAAGTAACCATCAAAGCGG-3’), which gave a 681 bp product only in the targeted allele (not shown). Hereafter, we refer to cells or mice homozygous for the targeted allele (i.e., genotype *Ocstamp* ^*tm1a(KOMP)Wtsi/tm1a(KOMP)Wtsi*^) as “OCSt-KO,” for OC-STAMP knockout.

### Skeletal analysis

X-rays were obtained as previously described using a Faxitron X-ray cabinet [18]. For 6-week old mice, pQCT for volumetric bone mineral density and micro-CT for trabecular bone values were performed as described previously [19]. For 2-week-old animals, only micro-CT was performed because the amount of mineral is too low for pQCT analysis. Briefly, femora were collected at 2 and 6 weeks, fixed in 3.7% formaldehyde at 4° for about 1 week, and then stored in cold 95% ethanol until analyzed. Femur length was measured with digital calipers (Stoelting, Wood Dale, IL). Then, isolated femora were assessed using the pQCT system from Stratec XCT-RESEARCH (Norland Medical System, Fort Atkinson, WI), operating at a resolution of 0.07 mm. For micro-CT, scans were performed with a MicroCT40 instrument (Scanco Medical AG, Bassersdorf, Switzerland) to evaluate trabecular bone volume fraction (BV/TV) and micro-architecture in the metaphyseal region of the distal femur. In addition, cortical thickness data were obtained at the mid-shaft. Group sizes analyzed at 2 weeks were n = 15 for genotype ^+/+^, n = 11 for OCSt-KO; at 6 weeks, n = 12 for ^+/+^, and n = 15 for OCSt-KO. Parameters measured included bone volume/total volume; bone area/total area; medullary area/total area; total density; cortical area; trabecular area; trabecular density; trabecular content; trabecular spacing; and connectivity density.

The contralateral femur was dissected, fixed, embedded in glycol methacrylate (Polysciences, Warrington, PA), sectioned at 3 μm, and stained for tartrate-resistant acid phosphatase (TRAP) activity as previously described [20–22]. Two observers evaluated at least 3 TRAP-stained sections from 3 animals of each genotype (WT and OCSt-KO), with and without toluidine blue counterstain, to determine the presence or absence of multinucleated, TRAP-positive osteoclasts. Images were obtained using a Zeiss Axioskop 2 and a Zeiss Axiocam HRc digital camera using Zeiss Axiovision 4.8.1 (Carl Zeiss, Thorwood, NY). No alterations to any images were done except for exposure settings and total image brightness and contrast. For image analysis of TRAP-positive area, at least 3 sections of femoral distal metaphysis from at least 3, 6-week-old mice of each genotype were obtained using a 5× objective at 3900 × 3300 pixels. TRAP enzyme histochemistry was developed and stopped on all slides simultaneously in one batch. Images were analyzed using Photoshop 7.0 (Adobe, Systems, San Jose, CA). TRAP-positive areas were selected and quantified by color-matched thresholding. Thresholded images were then made binary (black and white), and the resulting binary images were analyzed by NIH ImageJ for total TRAP-positive area (μm^2^), mean TRAP-positive cell size (μm^2^), and fraction of total image which is TRAP-positive. Results for each genotype were pooled for analysis by ANOVA (Microsoft Excel 2010).

### HEK cell cultures and transient transfections

HEK 293 and 293T cells (ATCC, Manassas, VA) were maintained in Dulbecco’s modified Eagle's medium (DMEM; Life Technologies, Carlsbad, CA) supplemented with 10% FBS (Sigma, St. Louis, MO), 1% penicillin/streptomycin (Life Technologies) in a humidified incubator at 37°C in 5% CO_2_. Transfection of cells was done using Lipofectamine 2000 (Life Technologies) and was performed according to the manufacturer’s instructions.

### Molecular cloning and mutagenesis

All plasmids and viral constructs were verified by sequencing. Mouse OC-STAMP cDNA (accession number NM_029021) was amplified from the pcDNA3.1D/V5-His-TOPO expression vector previously described (Yang et al., 2008) and cloned into pLenti-CMV-MCS-SV-Puro viral vectors (a generous gift of Dr. Hong Zhang, University of Massachusetts Medical School, Worcester, MA) to generate pLenti-CMV-OC-STAMP vectors. eGFP sequence was C-terminally cloned into the pLenti-CMV-OC-STAMP vector (pLenti-CMV-OC-STAMP-GFP vector). The OC-STAMP cDNA was also cloned into the p3XFLAG-myc-CMV-26 expression vector (Sigma) in-frame, with the triple FLAG tag at the N-terminus. The glycosylation-deficient mutation, N162D, was produced using the QuikChange XL site-directed mutagenesis kit (Stratagene, LaJolla, CA), to replace A 548 in the mouse cDNA with G, thereby changing the AAT asparagine codon 162 to a GAT aspartic acid codon. Anti-GFP antibody produced in rabbits was purchased from Life Technologies.

### Glycosylation analysis

HEK cells were transiently transfected with either wild type OC-STAMP or OC-STAMP N162D in the pcDNA-V5-His-Topo vector. After 24 hr of expression, cells were lysed in SDS-PAGE sample buffer containing protease inhibitors (Protease Arrest, G-Biosciences, St. Louis, MO). Deglycosylation reactions were performed with N-glycanase (PNGase F, New England Biolabs, Ipswich, MA) in G7 buffer with NP-40 at 37°C for 1 hr according to the manufacturer’s instructions. Samples were analyzed by SDS-PAGE, blotted onto PVDF, and probed with anti-V5-HRP conjugate (Life Technologies, #R96125).

### Osteoclast differentiation in vitro

Femora and tibiae were dissected from mice between 3 and 6 weeks of age. Bone marrow cells were flushed out with α-MEM and cultured in Petri dishes in α-MEM containing 10% FBS and 75 ng/ml M-CSF (recombinant human CSF-1, Chiron Corp., Emeryville, CA); [6]. After 4 days, adherent cells were used as bone marrow mononuclear cells (BMMCs). The cells were plated in 48-well plates at a density of 15, 000 cells/well and cultured in the presence of 10 ng/ml mouse RANKL (R&D Systems, Minneapolis, MN) and 75 ng/ml M-CSF (Chiron Corp., Emeryville, CA) for 4–6 days. The medium was replaced every 2 days.

### Lentiviral transduction

Recombinant lentiviruses were produced as previously described [23]. Briefly, either pLenti-CMV-GFP, pLenti-CMV-OC-STAMP-GFP, or pLenti-CMV-OC-STAMP N162D-GFP lentiviral plasmids were co-transfected with psPAX2 and pMD2.G into HEK293T cells using Lipofectamine 2000. The medium was replaced the following day, and viral supernatants were harvested 48 hours post-transfection. Primary BMMCs isolated from WT and KO mice and cultured in Petri dishes for 2 days were transduced with lentiviruses in the presence of 8 μg/ml polybrene (Sigma). Infected BMMCs were selected with 2 μg/ml puromycin for 3 days.

### TRAP staining and TRAP activity assay

BMMCs were cultured under differentiation conditions for 6 days. Cells were rinsed with PBS and fixed with 4% paraformaldehyde for 20 min, and washed twice with PBS. Fixed cells were stained for TRAP using a Leukocyte Acid Phosphatase kit (Sigma). TRAP staining was performed in triplicate wells for each condition according to the manufacturer’s instructions. To measure secreted TRAP, cells were plated on a 96-well plate at a density of 6, 000 cells/well and cultured in the presence or absence of RANKL for 4 days. On day 4, 25 μl of the medium was mixed with 75 μl of TRAP solution. The mixture was incubated at 37°C for 2 hr and the absorbance was read at 540 nm (adapted from the protocol of a TRAP staining kit, B-Bridge International, Inc., Cupertino, CA, USA; [23]). For quantitation of osteoclast area, micrographs were obtained of each of 3 wells from each culture condition. The first 20 (±1) multinucleated cells (3 or more nuclei) observed in each image were outlined using Image J software, the area of osteoclast cell was measured, and the mean area per cell was calculated.

### In vitro resorption assay

BMMCs were seeded on 24-well Osteo Assay Plates (Corning, NY) at a density of 30,000 cells/well, and cells were differentiated in α-MEM containing 75 ng/ml M-CSF and 10 ng/ml RANKL for 6 days. The medium was replaced every 2 days. Cells were removed gently with 10% bleach and the wells rinsed with distilled water and air dried. The dried plates were scanned at 2400 pixels per inch on a flatbed scanner (Microtek 9800 XL). Only global adjustments of brightness and contrast were performed on the resulting grayscale images before they were thresholded and analyzed. The total resorbed pixel area in triplicate wells was analyzed by ImageJ software. The experiment was repeated 3 times.

### Actin ring formation on dentine discs

BMMC (30,000 cells/well) were seeded on dentine discs in 48-well plates. Cells were differentiated in medium containing 75 ng/ml M-CSF and 20 ng/ml RANKL and maintained for 9 days. The medium was replaced every 2 days. Cells were fixed with 4% paraformaldehyde for 20 min and washed twice with PBS. Fixed cells were permeabilized with 0.1% Triton X-100 in PBS for 15 min. After rinsing with PBS, cells on the dentine disc were incubated with Alexa Fluor 568 Phalloidin (1:100 diluted in PBS, Life Technologies) in the dark for 1 hr. The discs were washed and placed on glass slides and mounted with 70% glycerol. Actin rings were visualized with a Zeiss Axiovert 40 CFL fluorescence microscope.

### ELISA Assays

Blood was obtained from anesthetized mice by cardiac puncture at 6 weeks of age, and sera were stored in aliquots at -80°C. All ELISAs were performed as instructed by the manufacturer. C-terminal telopeptide (CTX) measurements were performed using the RatLaps ELISA (Immunodiagnostic Systems, Gaithersburg, MD; #AC-06F1). TRAcP 5b protein measurement was performed using Mouse TRAP ELISA (Immunodiagnostic Systems; #SB-TR103). Osteocalcin measurement was performed using the Mouse Osteocalcin EIA kit (Biomedical Technologies, Ward Hill, MA # BT-470). Assays were run in triplicate on between 3 and 9 individual animals per genotype.

### Digitonin treatment and immunofluorescence

HEK293 cells grown on glass coverslips were fixed with 3% paraformaldehyde in PBS for 10 min. and washed in PBS. For selective permeabilization of the plasma membrane, cells were incubated in buffer S (10 mM HEPES-KOH, pH 7.4; 0.3 M sucrose; 0.1 M KCl; 2.5 mM MgCl_2_; 1 mM EDTA, and 50 μg/ml digitonin (Sigma)) for 3 min on ice [24]. For permeabilization of all membranes, cells were incubated with 1% Triton X-100 in PBS for 10 min and blocked with 1% BSA in PBS. Mouse anti-FLAG M2 antibody (1:500, Sigma, #A8592) and rabbit anti-GFP antibody (1:200, Life Technologies, #A11122) were diluted in blocking solution and incubated for 1 hr. at room temperature. Primary antibody binding was visualized using fluorescent dye-conjugated secondary antibodies: goat anti mouse Alexa 568 (Life Technologies, #A11032) or goat anti rabbit Alexa 568 (Life Technologies, #A11036) incubated for 45 min. DAPI (Life Technologies) staining was used to visualize nuclei. The samples were mounted with ProLong Gold antifade reagent (Life Technologies) and visualized using inverted fluorescence microscopy (Leica DMI6000) or a Leica SP5 (II) laser scanning confocal microscope (Leica, Buffalo Grove, IL) equipped with 40X (1.30 NA) and 63X (1.4–0.6 NA) oil immersion lenses. Leica LAS AF Lite software was used for recording and image processing.

### Cell viability

Cell viability was assessed using the MTT kit (Cayman Chemical Company, Ann Arbor, MI) according to the manufacturer’s instructions. Each condition was measured in triplicate.

### RNA isolation and quantitative RT-PCR

BMMCs were seeded at a density of 250,000 cells/well in 6-well plates. Cells were treated with 75 ng/ml M-CSF and 10 ng/ml of RANKL in complete α-MEM for 3 and 6 days with medium changed every two days. Total RNA was extracted using the RNeasy kit (Qiagen, Germantown, MD). One μg of RNA was reverse transcribed to cDNA using the QuantiTect Reverse Transcription Kit (Qiagen) according to the manufacturer’s protocol. The PCR reactions were performed in triplicate using QuantiFast 2X SYBR Green PCR Master Mix (Qiagen) in a LightCycler 2 system (Roche Diagnostics, Indianapolis, IN) as follows: 95°C for 5 min, then 40 cycles of 95°C for 10sec and 60°C for 30 sec. Δ*C*_t_ for each gene was calculated and represents the difference between the *C*_t_ value for the gene of interest and the *C*_t_ value for the reference gene (*Rplpo*). Fold-changes were calculated using the 2^*−*ΔΔ*C*^_t_ convention. Primer sequences are shown in **S1 Table**.

### Statistical tests

In vitro experiments were repeated at least 3 times with at least triplicate determinations for each condition in each experiment, except for the MTT assay which was performed twice in triplicate. For all in vitro data, statistical analyses were done by ANOVA (GraphPad Prism 6 or Microsoft Excel) or by two-tailed *t*-tests following *F*-tests to determine whether to treat variances as equal or unequal (Microsoft Excel 2010). In both cases, *P* < 0.05 was considered significant. pQCT and micro-CT analysis was done by ANCOVA using MP version 6.0 software (SAS, Cary, NC), as described [19].

## Results

### Mice

Mice carrying the “knockout first” targeted allele were bred and maintained in the C57BL/6J background. The targeted allele is shown in **Fig 1A**. PCR genotyping yielded the expected band sizes (**Fig 1B**). Correct insertion was further verified by PCR of the 3’ end of the insert (not shown). We noted that this produces an effective knockout, so further crosses with either flippase- or Cre- expressing mice were not performed. Those recombination sites are in place for future studies of targeted deletions, as needed. Knockout mouse consortium standard nomenclature for the targeted allele is *Ocstamp* ^*tm1a(KOMP)Wtsi*^. For the rest of this report, we refer to mice homozygous for the allele, genotype *Ocstamp* ^*tm1a(KOMP)Wtsi/ tm1a(KOMP)Wtsi*^, as “OCSt-KO” for OC-STAMP knockout. Radiographs showed no evident bone abnormalities at either 2 (**Fig 1C**) or 6 weeks (not shown). More detailed skeletal analysis of dissected, fixed femora by micro-CT and pQCT was also done. Those analyses found no statistically significant differences between wild type and OCSt-KO mice for any micro-CT (2- and 6-week samples) or pQCT (6-week samples) parameters measured at either age. Excel files containing the pQCT and micro-CT original data are attached as **S2 and S3 Tables**, respectively, and image files of the statistical analyses are attached as **S1, S2 and S3 Figs.** Note that bones from 2-week-old mice have too little mineral to obtain data from pQCT.

**Fig 2:**
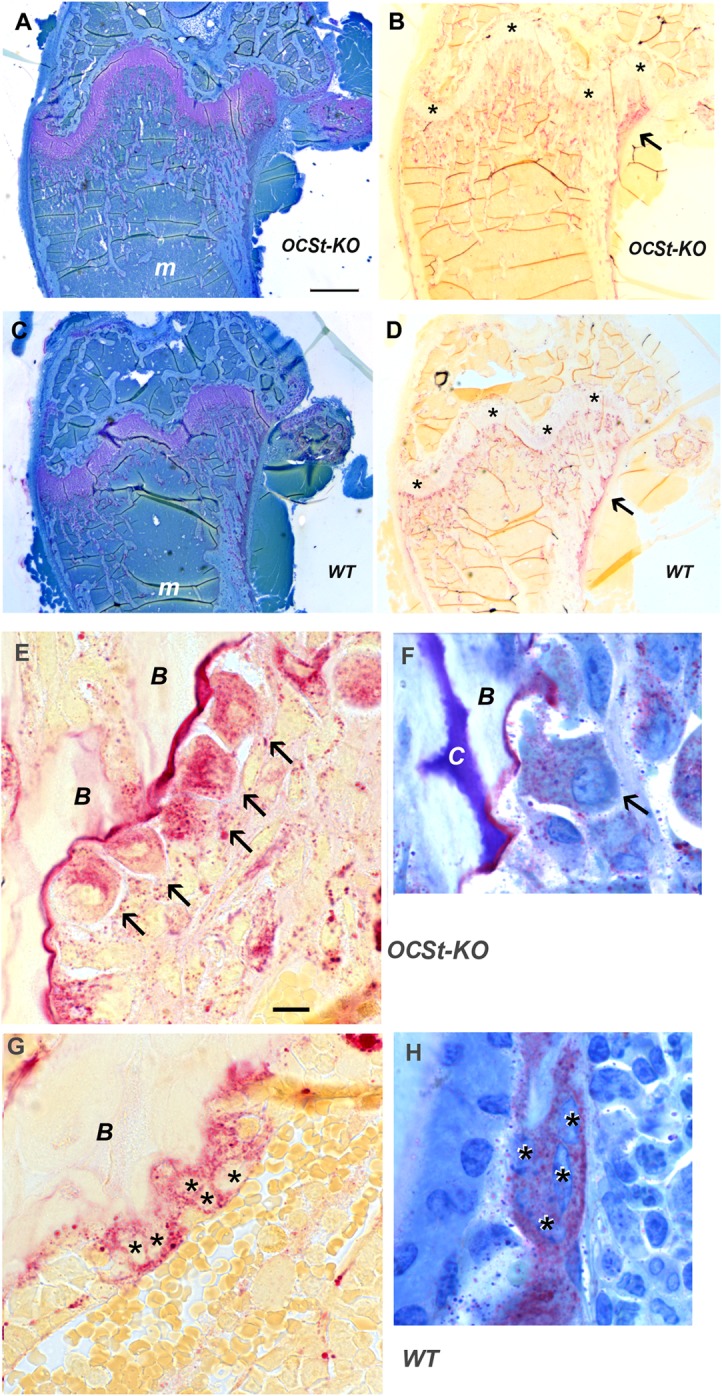
Low and high power histology of adjacent sections (A, B and C, D) of OCst-KO (A, B, E, F) and WT (C D, G, H) 6-week-old mouse distal femur. Glycol methacrylate, 3 μm sections were stained histochemically for TRAP (**B, D, E, G**) and some were counterstained with toluidine blue (**A, C, F. H**). At low power, overall appearance was highly similar, with growth plates normal (purple, wavy band in **A** and **C,** asterisks in **B** and **D**), similar trabecular size and thickness in both the primary and secondary ossification centers, and open marrow space (*m*) in the diaphysis. TRAP stains (**B, D**) show overall similar distribution of osteoclasts, including along the chondroosseous junction, among the trabeculae of the primary ossification center, and notably, along the periosteal surface (arrows in B and D), where the bone is being removed to maintain the flared shape of the metaphysis. At higher power (**E, F, G, H**), only mononuclear osteoclasts are present in OCSt-KO mice. A row of such cells is indicated in **A** by arrows, attached to trabecular bone (*B*) in the primary spongiosa. In contrast, wild type mice had typical, multinucleated osteoclasts in this area, and individual nuclei (visible by lack of overlying TRAP label in **C**), are indicated by asterisks. Blinded observers consistently identified OCSt-KO and WT sections based solely on the presence or absence of multinucleated osteoclasts. Bar in A = 500 μm in A, B, C, D. Bar in E = 10 μm in E and G; 7.3 μm in F and H. *B* = bone, *C* = cartilage core.

**Fig 3:**
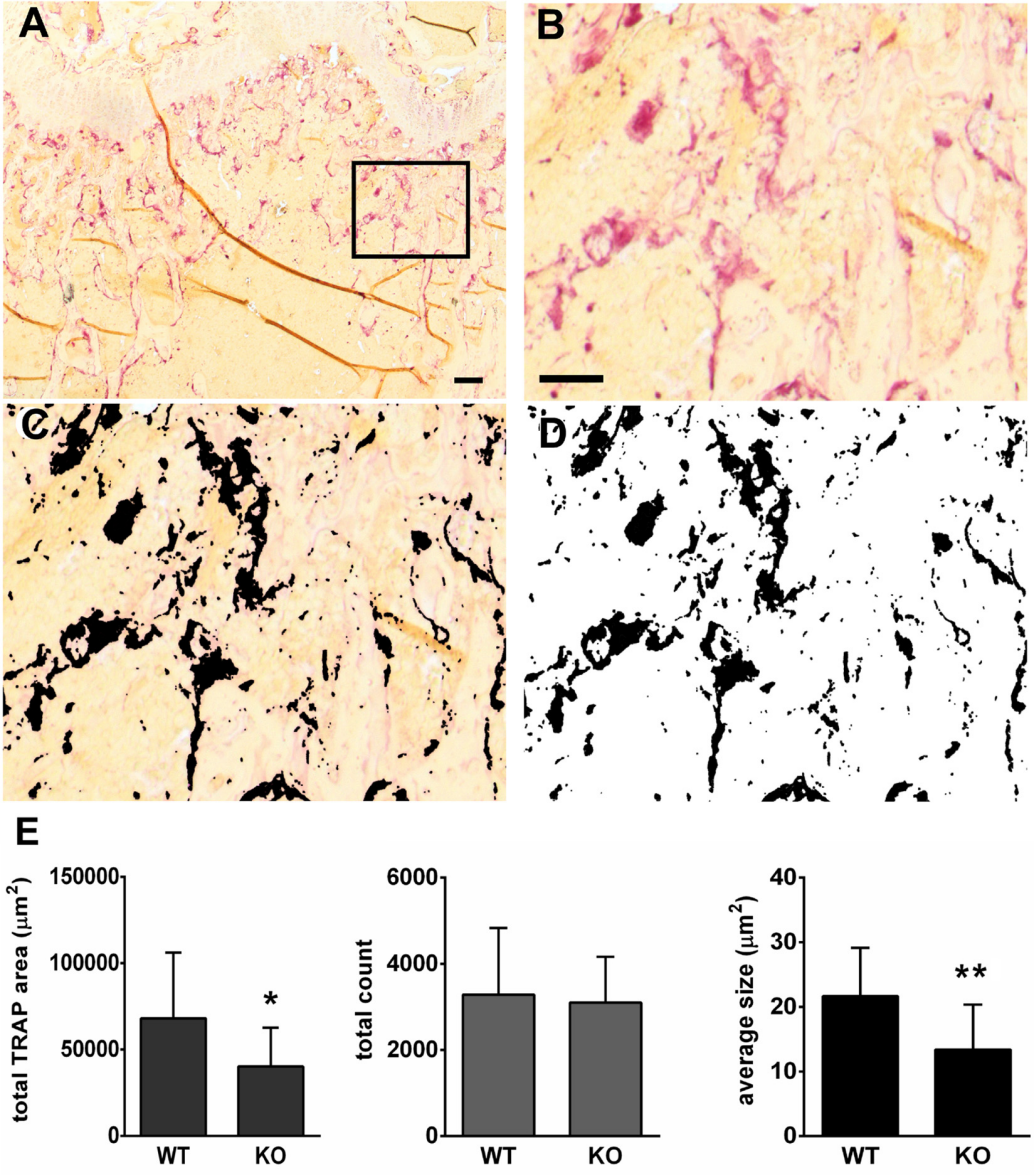
TRAP-positive osteoclast measurements on bone sections. Images were obtained with a 5X objective of distal femora of WT and OCSt-KO mice at 6 weeks post-partum and TRAP-positive total area, number of TRAP-positive sites, and mean area of those sites were measured. **A**, A typical whole image of distal femur metaphysis, stained histochemically for TRAP (red) without counterstain. The region outlined in **A** is enlarged in **B. C**. shows the same area of the section after color matching was performed to select TRAP-positive sites in the section (now black). Finally, the images were made binary (**D**), leaving only TRAP-positive (black) and TRAP-negative (white) areas for analysis. Bar in A = 100 μM, bar in B = 50 μm in B, C, and D. E. The mean total TRAP-positive area per micrograph differed significantly between WT and OCSt-KO (left; *P< 0.05). The total count of TRAP-positive sites was not different between genotypes (middle); however, the mean area of each TRAP-positive sites was significantly lower in OCSt-KO (*P < 0.02). Results from 3 or 4 sections per animal, and 3 animals per genotype were pooled for analysis.

Histological evaluation of TRAP-stained sections of femora showed overall similarity between OCSt-KO and WT at both 6 weeks (**Fig 2A–2D**) and 2 weeks (not shown), consistent with the micro-CT and pQCT results. Organization of bones and the disposition of TRAP-positive osteoclasts were similar between genotypes, with growth plates appearing normal, and with osteoclasts plentiful along the chondroosseous junction, among the trabeculae of the primary and secondary ossification centers, and with high TRAP activity evident along the periosteal surface where the flared metaphysis was being shaped. Higher magnification (**Fig 2E–2H**) revealed that only mononuclear osteoclasts were present in OCSt-KO mice, and blinded observers routinely distinguished OCSt-KO sections from wild type by that criterion alone (n = at least 3 sections from at least 3 animals per genotype for 2 blinded observers). Quantitative image analysis was performed to assess the total TRAP-positive area, the number of TRAP-positive spots, and the mean size of the TRAP-positive spots per section in at least 3 sections from 3 animals at 6 weeks old **(Fig 3).** We found significant differences between genotypes (WT *vs*. OCSt-KO) in total TRAP-positive area per section and mean area per TRAP-positive spot. TRAP-positive total area per micrograph was 67,122 ± 37,967 μm^2^ in WT (n = 11 micrographs analyzed) and 40,192 ± 22,582 μm^2^ in OCSt-KO (n = 11 micrographs analyzed), *P <* 0.05. The mean size of the TRAP-positive spots was 21.6 ± 7 μm^2^ for WT and 13.4 ± 6.9 μm^2^ for OCSt-KO (*P*< 0.02). This is consistent with smaller size of mononuclear osteoclasts. We did not find a significant difference in the number of TRAP-positive spots per section. Values were 3,282 ± 1,546 for WT and 3,100 ± 1,061 for OCSt-KO, *P* > 0.05. Together, this suggests that there were roughly equivalent numbers of osteoclasts, that the mean area per cell was smaller in the OCSt-KO (consistent with their mononuclear state), and that the total TRAP-positive area was also slightly lower in the knockouts.

### Bone metabolic marker analysis

**Fig 4:**
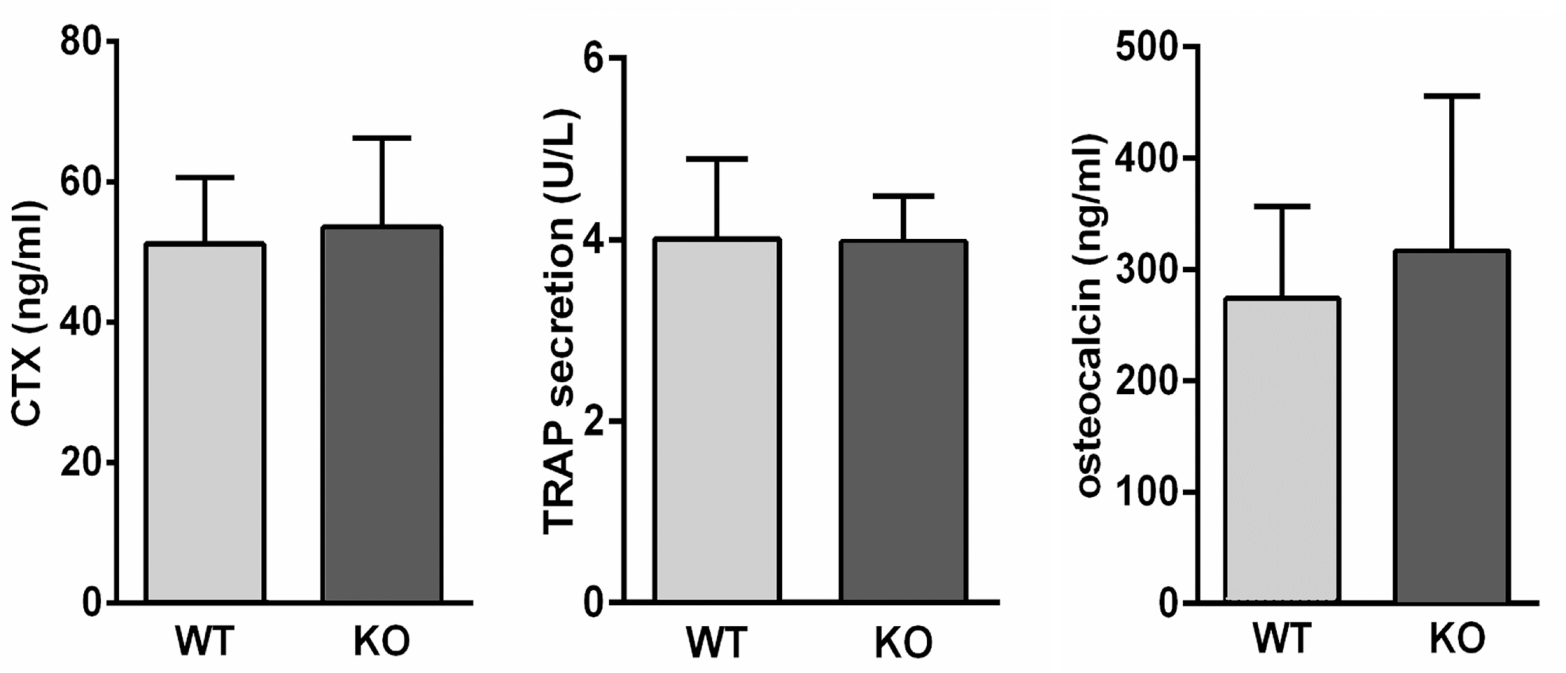
Bone formation/resorption indicators were normal in OCSt-KO mice. Serum levels of bone resorption markers collagen C-terminal peptides (CTX, left), and TRAP (middle), as well as the bone formation marker, osteocalcin (right), were measured in OCSt-KO and WTmice at 6 weeks post-partum. No significant differences in any of these parameters were found, consistent with normal rates of bone resorption and formation. Triplicate assays were run on samples from 9 (CTX), 5 (TRAP), and 9 (osteocalcin) WT mice, and on 4 (CTX), 3 (TRAP), and 3 (osteocalcin) OCSt-KO mice.

Previous work had shown that OC-STAMP knockout osteoclasts had a roughly 5-6-fold lower ability to resorb bone in culture [14]. Given the normal bone architecture and mineral density, we evaluated whether bone turnover markers would indicate any differences between WT and OCSt-KO mice. To this end, we performed ELISA assays for osteoclast activity markers CTX and TRAP, and for the osteoblast activity marker, osteocalcin, all measured in triplicate on serum from 6-week-old mice. We found no significant differences for any of the three markers between genotypes **(Fig 4A–4C)**. CTX levels were 51.1 ± 1.8 ng/ml in WT (n = 9 animals) and 53.6 ± 3.6 ng/ml in OCSt-KO mice (n = 4 animals). TRAP (TRAcP 5b) values were 4.0 ± 0.2 U/L for WT (n = 5 mice) and 3.9 ± 0.1 for OCSt-KO (n = 3 mice). For osteocalcin, the values were 273.9± 21.2 for WT (n = 5 mice) and 316.7 ± 46.2 for OCSt-KO (n = 3 mice), *P* > 0.05 for all 3 analytes. In sum, there were no measurable differences between genotypes in bone formation or bone resorption markers.

### Defective fusion of OCSt-KO osteoclasts is rescued by OC-STAMP lentivirus

**Fig 5:**
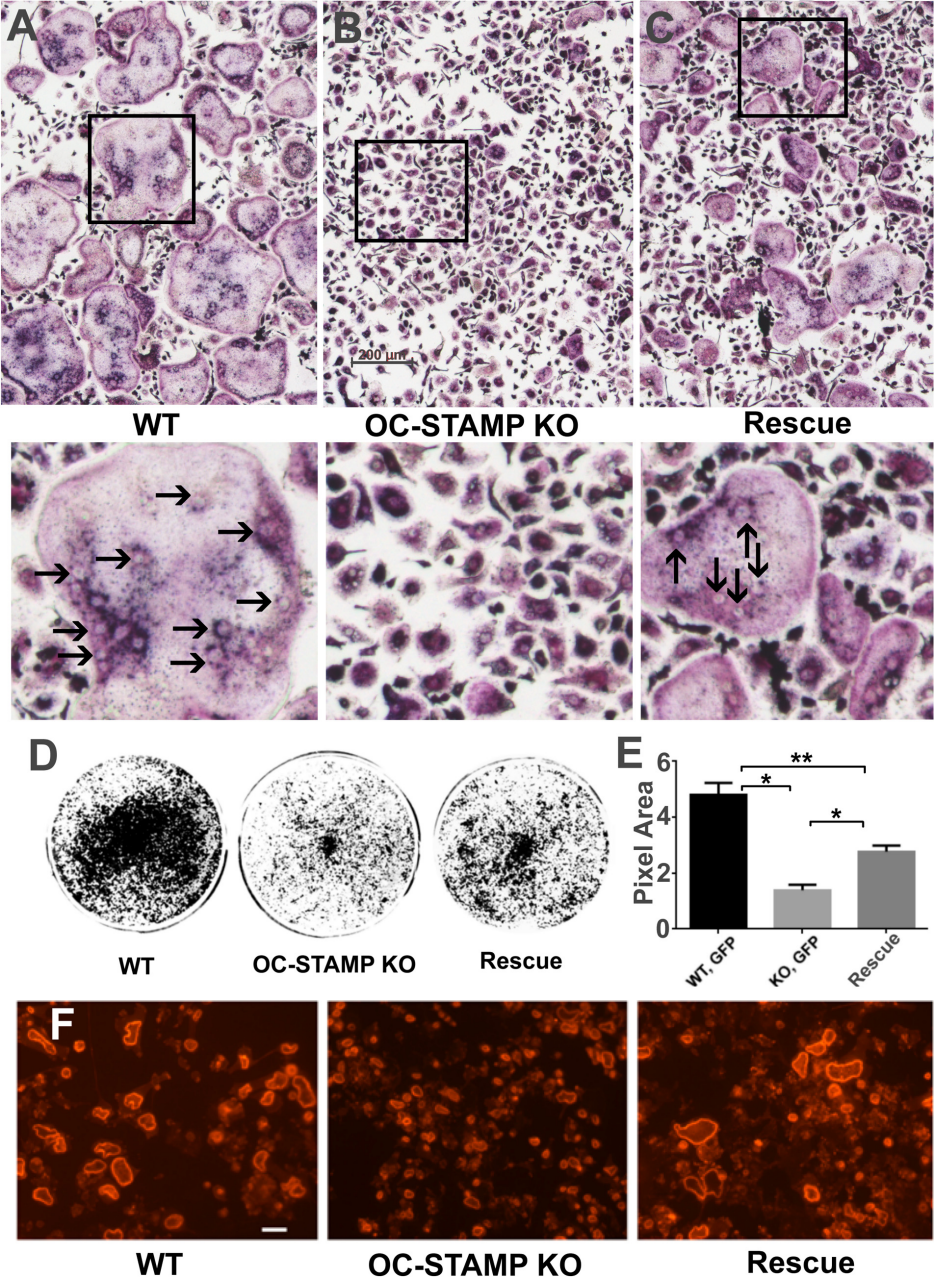
Rescue of OCSt-KO BMMC by lentiviral transduction. TRAP stain shows BMMC from WT mice transduced with GFP (**A:** WT), from OCSt-KO mice transduced with GFP (**B**: OC-STAMP KO), and from OCSt-KO mice transduced with OC-STAMP:GFP (**C**: Rescue) following 6 days of culturing in the presence of RANKL. Many large, multinucleated osteoclasts are seen in WT cells, whereas none are present in the KO cells. Fusion is rescued by transduction with OC-STAMP. Scale bar in B = 200 μm. Boxed areas in **A, B**, and **C** upper panels are shown at higher magnification below. Some individual nuclei within multinucleated cells are indicated by arrows in the WT and Rescue panels. **D.** BMMC were transduced and cultured as above on HA coated plates, and the plates were scanned after 6 days of culture. Resorbed HA appears as black. **E.** Quantitation of resorbed area shows a roughly 3.5-fold decrease of resorption activity in KO *vs*. WT, whereas the rescued cells had their activity mostly restored. Mean + s.d. is shown, n = 3, *P < 0.002; **P < 0.005. **F.** TRITC-labeled phalloidin shows large actin rings on dentine disks in WT (left) and rescued (right) cells. KO mononuclear osteoclasts (middle) also made actin rings, but they were much smaller, consistent with small cell size. Scale bar in F = 100 μm.

**Fig 6:**
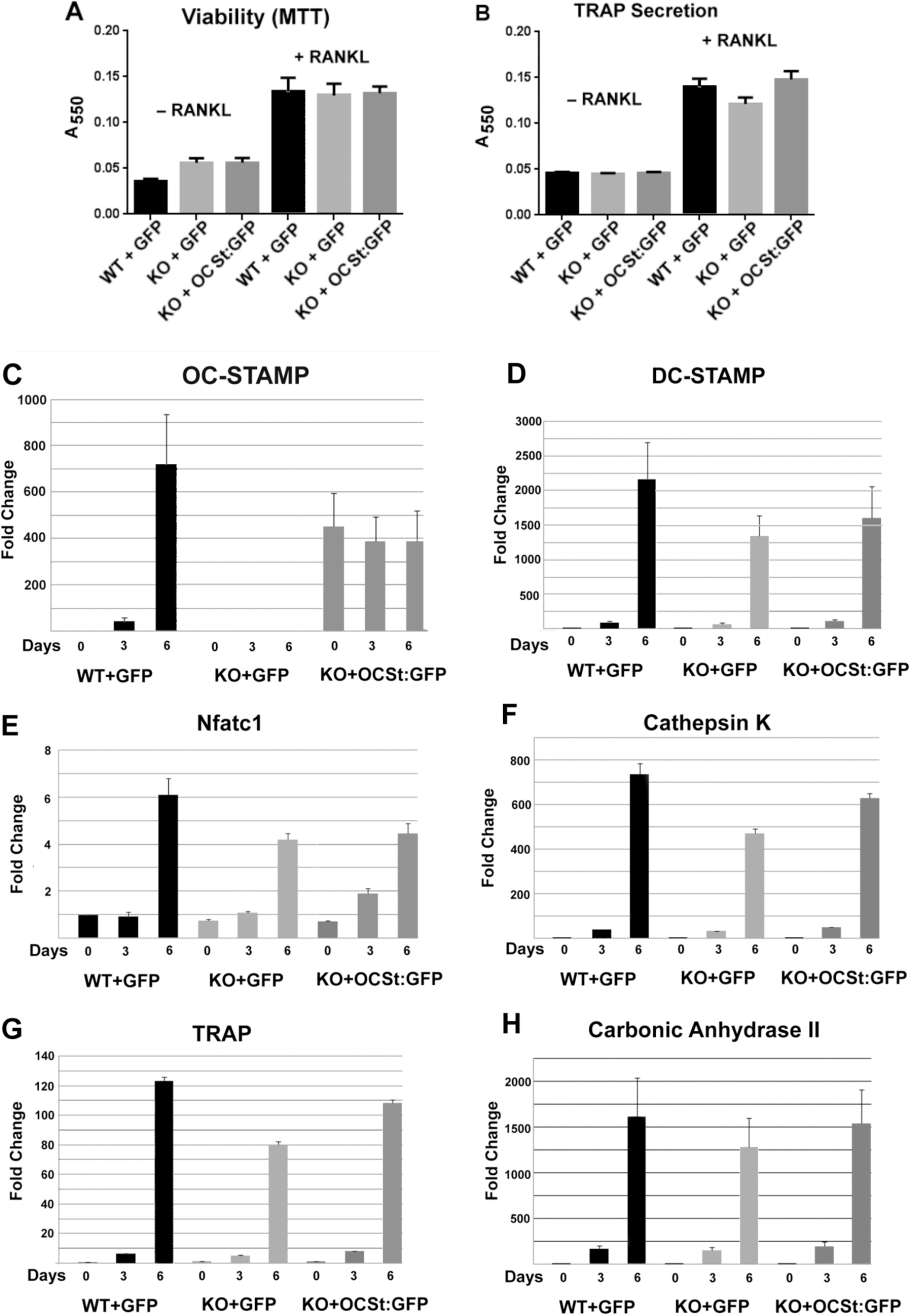
Viability, secretion, and gene expression. **A.** Viability. BMMC from wild type (WT) or OCSt-KO (KO) were virally transduced, cultured with or without RANKL for 6 days as indicated, and MTT assays for cell viability were performed. Transduction with either GFP or OC-STAMP-GFP (OCSt:GFP) lentivirus had no effect on viability. Mean + s.d., n = 3. **B.** TRAP secretion. Supernatants from cells cultured as in **A** were analyzed for secreted TRAP enzyme. No significant difference in TRAP secretion was seen between wild type (WT) and OCSt-KO (KO) cells, whether transduced with GFP or with OC-STAMP fused to GFP (OCSt:GFP). Mean + s.d., n = 3. **C-H.** mRNA expression of osteoclast markers. Primary BMMC cells were virally transduced and were cultured for 0, 3, and 6 days in the presence of RANKL, and Q-PCR analyses for osteoclast mRNAs were performed, as indicated. Note that no OC-STAMP mRNA was detected in OCSt-KO (KO) cells at any time point unless the viral vector encoded OC-STAMP (OCSt:GFP), whereas no other osteoclast markers were significantly affected; n = 3.

BMMCs were transduced with either GFP or OC-STAMP:GFP lentivirus, selected with puromycin, and differentiated with RANKL for 6 days. Expression of GFP and OC-STAMP:GFP protein in BMMCs was confirmed by western blot.Virally transduced cells treated with RANKL for 6 days were stained for TRAP. As shown in **Fig 5A**, large, TRAP-positive, multinucleated osteoclasts were formed from WT BMMCs, whereas there were only TRAP-positive mononuclear osteoclasts from OCSt-KO BMMCs (**Fig 5B**), indicating complete failure of osteoclast precursors to fuse. This fusion defect was rescued by expression of OC-STAMP (**Fig 5C**). Higher magnifications of boxed areas are also shown, and some of the multiple nuclei in large osteoclasts are indicated by arrows in the WT and rescue samples. Osteoclast area was averaged for approximately 60 consecutive multinucleated (3 or more nuclei) cells in digital micrographs of each conditions. Wild type mean osteoclast area was 45,399 ± 7230 μm^2^; n = 58. OCSt-KO cells had zero multinucleated cells. Virally rescued OCSt-KO osteoclasts (transduced with OC-STAMP:GFP) had a mean osteoclast area of 18,536 ± 2054 μm^2^; n = 60; *P*< 0.0005 comparing WT to OC-STAMP:GFP rescue (**S4 Table**). The level of rescue, i.e., mean area of multinucleated osteoclasts, was significantly lower than WT, corresponding well to the degree of OC-STAMP mRNA expression (see Q-PCR, **Fig 6C**). In sum, replacing the deleted OC-STAMP was necessary and sufficient to restore fusion of OCSt-KO precursors during osteoclast differentiation, and the degree of rescue is consistent with the OC-STAMP mRNA level achieved by viral transduction.

We next investigated the effects of OC-STAMP:GFP protein expression on resorptive activity using hydroxyapatite (HA)-coated plates. As shown in **Fig 5D and 5E**, OCSt-KO osteoclasts were capable of resorbing HA, but at a roughly 3.5-fold lower rate than their wild type counterparts (4.81 *vs*. 1.39 pixel area, *P*< 0.002). This resorption defect was significantly improved by transduction with OC-STAMP:GFP. OCSt-KO cells transduced with OC-STAMP:GFP lentivirus resorbed a statistically significantly greater area of HA compared to OCSt-KO osteoclasts infected with GFP alone (2.77 *vs*. 1.39 pixel area, *P*< 0.002). Thus, restoring expression of OC-STAMP in OCSt-KO BMMCs significantly increased not only fusion, but also the resorbing activity of osteoclasts.

We also evaluated the appearance of rescued cells compared to wild type on dentine discs, a more physiological substrate than HA plates, with both protein and mineral present. Actin rings were visualized by phalloidin labeling, and, as shown in **Fig 5F**, OCSt-KO cells formed very small actin rings compared to WT osteoclasts (compare left and middle panels), whereas OCSt-KO osteoclasts expressing OC-STAMP:GFP protein generated large actin ring structures (**Fig 5F,** right panel), consistent with restoration of fusion and osteoclast activity. In sum, viral replacement of OC-STAMP in OCSt-KO cells restored fusion, resorption, and actin ring size in the various experimental conditions tested.

### Cell viability, secretory activity and gene expression in OCSt-KO and rescued osteoclasts

WT and OCSt-KO BMMC were transduced with either GFP or, for OCSt-KO BMMC, with OC-STAMP:GFP lentivirus, and cultured with or without RANKL. **Fig 6A** shows that there was no differential effect of genotype or of transduction with GFP or OC-STAMP:GFP on viability, nor (**Fig 6B**) on the ability of the cells to secrete TRAP enzyme into the culture medium. **Fig 6C–6H** shows Q-PCR results for various osteoclast markers in those cells, with or without RANKL. Consistent with earlier reports [14,15], expression of OC-STAMP mRNA in WT BMMCs transduced with GFP alone was highly induced by RANKL and was also evident when OCSt-KO BMMC cells were transduced with OC-STAMP:GFP, whereas no OC-STAMP mRNA was detected in the OCSt-KO cells even in the presence of RANKL (**Fig 6C**). Knockout of OC-STAMP had no significant effect on expression of the remaining osteoclast markers analyzed (DC-STAMP, NFATC1, cathepsin K, TRAP, carbonic anhydrase II, **Fig 6D–6H**), although there was a trend to lower expression, consistent with an earlier report [14].

### OC-STAMP localization, topology, and glycosylation

**Fig 7:**
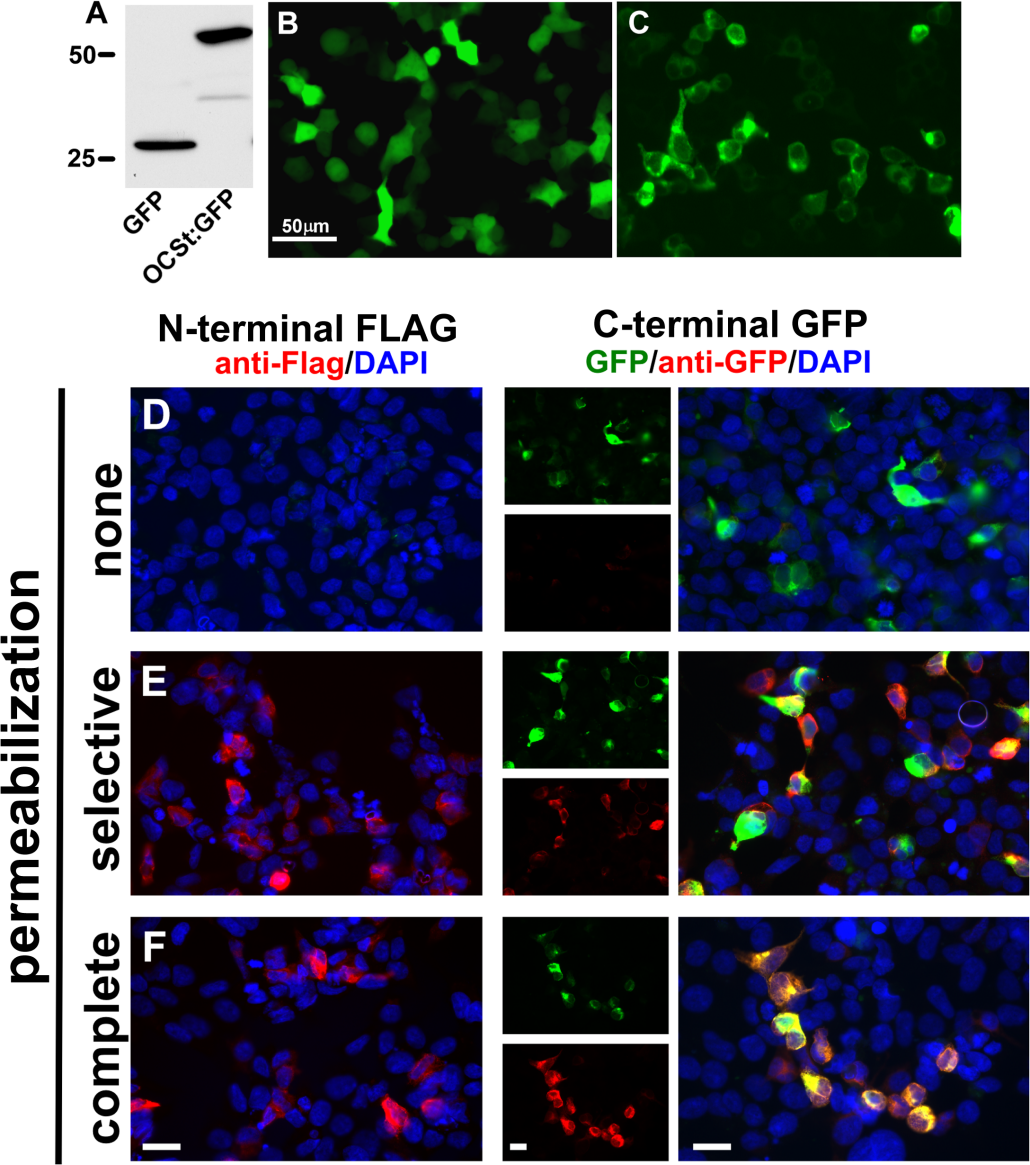
Peripheral localization of OC-STAMP and topology of N- and C-termini. **A-C.** HEK293T cells were transfected with either GFP or OC-STAMP-GFP vectors (OCSt:GFP). **A.** Western blot with anti-GFP antibody shows expected molecular weights (marker positions indicated at left). **B** and **C,** GFP fluorescence. In **B**, endogenous GFP fluorescence is seen throughout the cell, as expected for a cytoplasmic protein. In **C**, OC-STAMP-GFP signal is mainly confined to the cell periphery, as expected for a plasma membrane-localized transmembrane protein. Scale bar = 50μm. **D-F.** HEK 293 cells were transfected with tagged constructs indicated at top, permeabilized as indicated at left (none, plasma membrane selectively with digitonin, or all membranes with Triton X-100), and probed with anti-tag antibodies. With membranes intact, anti-FLAG antibody could not access the N-terminus, nor could anti-GFP access the C-terminus, whereas both antibodies could access their antigens when the plasma membrane or all membranes were permeabilized. For the GFP tag, separate green and red channels are also shown in small panels. GFP endogenous signal (green) vs. that for anti-GFP (red) varied in intensity in some cells, but the same cells were positive for both. Scale bars = 10 μm.

To establish OC-STAMP’s plasma membrane localization, HEK293 cells were transduced with either GFP lentivirus or OC-STAMP:GFP lentivirus (with GFP fused in-frame to the C-terminus of OC-STAMP) and cultured for 24h. There is no expression of endogenous OC-STAMP in HEK cells (data not shown). Transfected HEK cells were either lysed and analyzed by western blot (**Fig 7A**) or fixed and visualized by fluorescence microscopy (**Fig 7B and 7C**). As shown in **Fig 7A**, GFP protein was detected at the expected electrophoretic mobility (approximately 25 kDa) and the OC-STAMP:GFP fusion protein was present at approximately 67 kDa. In **Fig 7B**, GFP was present throughout the cell volume, while the OC-STAMP:GFP fusion protein was primarily localized at the cell periphery (**Fig 7C**). This is consistent with OC-STAMP being a transmembrane protein localized primarily to the plasma membrane. It is also consistent with the earlier observations that anti-OC-STAMP antibody inhibited fusion in vitro [15,16], i.e., that at least some parts of the protein are exposed on the cell surface.

To investigate the location of the N- and C-termini, which are critical to proper orientation of transmembrane proteins, we used OC-STAMP constructs carrying a 3X FLAG tag at the N-terminus or GFP at the C-terminus, then assessed the accessibility of the tags to antibody in cells with or without detergent permeabilization (**Fig 7D–7F**). Without permeabilization, no signal was detected using antibody to FLAG or GFP, and only the intrinsic green fluorescence of GFP was detected, indicating intracellular localization of both termini. Digitonin, which selectively permeabilizes the plasma membrane by extracting cholesterol [25], gave access by both antibodies to their respective tags and showed primarily peripheral distribution, consistent with plasma membrane localization and intracellular termini. Triton X-100, which non-selectively permeabilizes all membranes, also gave access to antibody (**Fig 7F**), and showed additional distribution of signal, consistent with some OC-STAMP being processed through the endoplasmic reticulum and Golgi, as expected for a cell surface protein. Together, these results show that both the N- and C-termini of OC-STAMP are intracellular, and support our original proposal based on transmembrane prediction algorithms [15].

**Fig 8:**
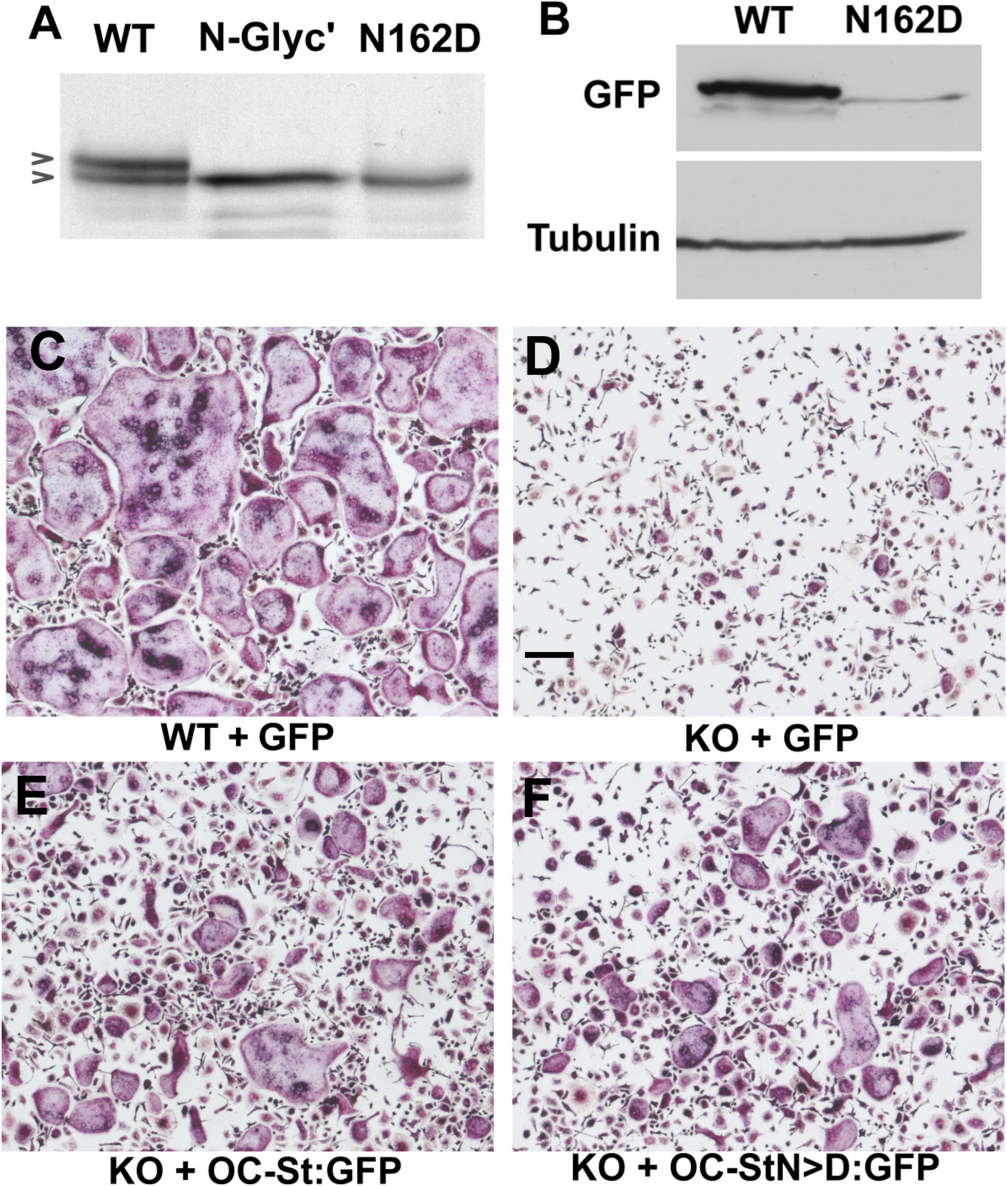
Glycosylation of OC-STAMP. **A**. HEK293 cells were transfected with V5-tagged wild type (WT) OC-STAMP or glycosylation-deficient (N162D) OC-STAMP. Wild type-transfected cell extracts were either untreated or digested with N-glycanase (N-Glyc’). Extracts were blotted and probed with anti-V5 antibody. Arrowheads indicate 2 bands, an upper, glycosylated form approximately 3kDa higher than the lower band at 50 kDa. Both N-glycanase-treated and the N >D mutation show only the low molecular weight form. Some of the overexpressed WT protein escapes glycosylation, giving 2 bands. **B**. BMMC from OCSt-KO mice were transduced with lentiviral vectors encoding wild type (WT) or (N162D) OC-STAMP fused to GFP. Cells were cultured for 6 days in RANKL, and extracts were blotted and probed with anti-GFP (upper panel) or anti-α-tubulin (lower panel). The glycosylated WT OC-STAMP appears to have much greater stability. **C**-**F.** TRAP enzyme cytochemistry. Wild type (WT) or OCSt-KO BMMCs were differentiated with RANKL for 6 days in 96-well plates. They were transduced with either GFP (**C, D**), OC-STAMP-GFP fusion protein (OCSt:GFP; **E**), or OC-STAMP-GFP fusion carrying the N162 mutation D (OC-StN>D; **F**). Fusion was rescued in the knockout cells by either the glycosylated or the non-glycosylated form of OC-STAMP. Bar in D = 200 μm.

To gain further details about OC-STAMP topology, we investigated a potential N-linked glycosylation site that is conserved in all mammals, birds, reptiles, and amphibians for which sequence data are available in GenBank. A representative list is shown in **Table 1**. This suggests that the loop containing the asparagine (N162 in mouse) resides on the extracellular side of the plasma membrane as predicted [15]. Its conservation also suggests that its function is important. To investigate this, we transfected HEK293 cells either with WT OC-STAMP or with OC-STAMP with N162 mutated to D (N162D). We also tested the effect of N-glycanase digestion on electrophoretic mobility. As shown in **Fig 8A**, WT OC-STAMP produced a doublet spaced about 3 kDa apart in western blots. This is consistent with OC-STAMP translated product being present in native and post-translationally glycosylated forms. N-glycanase treatment eliminated the upper band, indicating removal of a carbohydrate moiety. The N162D mutant OC-STAMP also produced only the lower band. Together, this shows that the loop containing N162 is glycosylated as predicted and therefore that it normally resides on the extracellular side of the plasma membrane.

**Table 1:** Conservation of glycosylation motif in terrestrial vertebrates.

Genus species	Alignment of glycosylation motif context	last aa
*Mus musculus*	QVLSCVTEGSLESLL**NTTY**QLRQAARELG-PASRAGSRSLTFEVEGK	**192**
*Bos taurus*	QVLRCVTEGSLESLL**NTTH**WLQTASQALN-PDGQAGSQGLTLQAQGD	**192**
*Ailuropoda melanoleuca*	RVLRCVTEGSLESLL**NTTH**QLHAASRALG-PAGHAGSQGLTLQAQGN	**192**
*Homo sapiens*	QVLRCVTEGSLESLL**NTTH**QLHAASRALG-PTGQAGSRGLTFEAQDN	**192**
*Monodelphis domestica*	QVLRCVAQGTLESLL**NSTQ**QLEATTEALDQAAGWAGGRRLTFETPGN	**182**
*Gallus gallus*	KVILCISKNSSESLL**NSTD**LLGNTFWKLEHELQ——NYLIWKPMDGHIQ	**196**
*Alligator mississippiensis*	QVIKCICKNSSESLL**NSTA**LLGSASWEFGHHIKPVFDSLLVWKPMNGPFQ	**193**
*Xenopus (Silurana) tropicalis*	RTLGCLSQHSSERLL**NSTF**FFQTMTSDTNDIVT-EMKNLLSSKKSD—VK	**179**

CLUSTALW2 alignment of OC-STAMP sequences from representative taxa show complete conservation of the Asp residue in a glycosylation motif (bold, underlined) and its amino acid context. It is present in rodents (*Mus musculus*), ungulates (*Bos taurus*), giant panda (*Ailuropoda melanoleuca*), primates (*Homo sapiens*), marsupials (opossum; *Monodelphis domestica*), birds (*Gallus gallus*), amphibians (*Xenopus (Siluriana) tropicalis*), and reptiles (*Alligator mississippiensis*). Alignments were performed of the whole protein sequences and the number of the last amino acid shown is listed on the right. The GenBank sequences used for the alignments were: gi|21312818, gi|297482088, gi|301787077, gi|225637556, gi|126303342, gi|118100617, gi|564236812, and gi|301607266, respectively.

To study effects of glycosylation on fusion of pre-osteoclasts, OCSt-KO BMMCs were transduced with either WT OC-STAMP:GFP or with mutant OC-STAMP (N162D):GFP lentivirus. As shown in **Fig 8B**, WT OC-STAMP:GFP produced a doublet in OCSt-KO BMMCs, consistent with the results in HEK293 cells (**Fig 8A**). As expected, the N162D mutant OC-STAMP produced only the lower band. Interestingly, the band intensity of mutant OC-STAMP was substantially lower than that of WT OC-STAMP, suggesting that glycosylation is important for protein stability. Next we tested whether changing N162 to D affected the function of OC-STAMP in the fusion of pre-osteoclasts. Both WT OC-STAMP:GFP and OC-STAMP (N162D):GFP rescued the defective fusion in OCSt-KO osteoclasts. Measurement of mean area of multinucleated osteoclasts, as described above, showed no significant differences between glycosylated and non-glycosylated OC-STAMP: WT: 48,313 ± 5090 μm^2^, n = 60; OCSt-KO rescued with OC-STAMP-GFP: 14,655± 1599 μm^2^, n = 61, *P<*0.0001 *vs*. WT; OCSt-KO rescued with OC-STAMP(N162D)-GFP: 10,827 ± 838 μm^2^, n = 60, *P<* 0.0001 *vs*. WT, *P* > 0.05 vs. OC-STAMP-GFP (**S4 Table**). Together, these results suggest that glycosylation is not essential for fusion under the conditions tested, but may be important for the stability of OC-STAMP protein.

**Fig 9:**
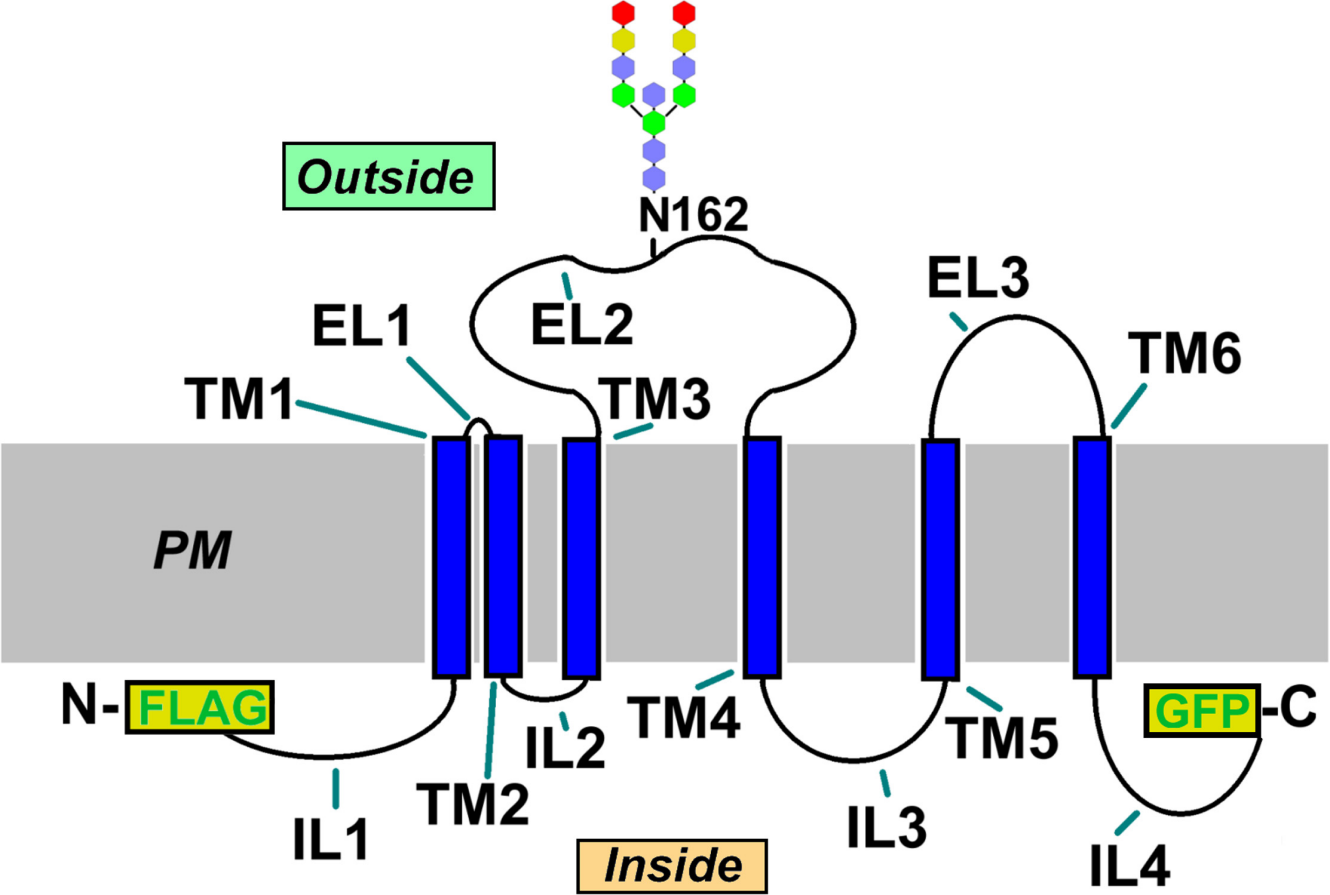
Diagram of OC-STAMP topology. Combining experimental results and topology predictions, the diagram shows current understanding of OC-STAMP topology with respect to the plasma membrane (PM). N- and C-termini are indicated, along with the position of the N-terminal FLAG and C-terminal GFP tags, and glycosylated residue N162. TM = transmembrane helix, IL = intracellular loop, EL = extracellular loop. A consensus mammalian core oligosaccharide is shown attached to N162: blue = GlcNac, green = mannose, yellow = galactose, red = Neu5Gc.

A model for OC-STAMP topology that is consistent with the extracellular glycosylation motif and intracellular termini data presented here and with the predictions we made in our original report [15] is shown graphically in **Fig 9. Table 2** lists the predicted internal, external, and transmembrane amino acid sequences for mouse OC-STAMP (Accession NP_ 083297.1).

**Table 2:** Topology of mouse OC-STAMP.

Feature	Orientation	aa #’s	Sequence
Intracellular loop 1	inside	1–24	MRTIRAATEHLFGLGWKFWRLGIC
Transmembrane helix 1	in to out	25–47	KAVVPLQAAWKAFSQPVPASCNE
Extracellular loop 1	outside	48–51	LLTQ
Transmembrane helix 2	out to in	52–74	LLLCVSLASLIAGLAHHWLVSLQ
Intracellular loop 2	inside	75–86	LYPLGPPALVTS
Transmembrane helix 3	in to out	87–109	LCGLFVFLSLGLVPPIRCLFVLS
Extracellular loop 2	outside	110–227	VPTLGSKQGRRLLLSYSAANLAVAVVPNVLGNVRAAGQVLSCVTEGSLESLL**N̳**TTYQLRQAARELGPASRAGSRSLTFEVEGKGSAFRLHMHTITQEILEDFSGLEFLARAALGTQRV
Transmembrane helix 4	out to in	228–250	VTGLFLLGLLGESAWYLHRYLTD
Intracellular loop 3	inside	251–300	LRFDNIYATRQLVRQLAQAGATHLLTSPPPWLLQTAQPKLSREELLSCLL
Transmembrane helix 5	in to out	301–323	RLGLLALLLVATAVTVASDYGAF
Extracellular loop 3	outside	324–398	LLAQAAVAWAQKLPTVPITLTVKYDASYKVLDFILFVLNQPPVESVFASMQRSFQWELRFTPHDCHLPQAQPPRV
Transmembrane helix 6	out to in	399–421	TAALAAGALQLLAGATLVLQAYA
Intracellular loop 4	inside	422–498	WRLRHTIAASFFPDQEARRLSHLQARLQRRHNQSDHLNKQPGTMATRESRKPGQGTRTLESQGPQAHDSLGPPYDLE

Transmembrane orientation of mouse OC-STAMP (GenBank accession # NP_083297.1) predicted by consensus of 5 algorithms utilized by the TOPCONS server (http://topcons.cbr.su.se/: SCAMPI-seq, SCAMPI-msa, PRODIV, PRO, and OCTOPUS). Also, we have shown in this report that the N- and C-termini are intracellular and that N162 (bold, underlined) is glycosylated, supporting those predictions.

## Discussion

Insertion of the selection cassette and associated targeting vector sequences into intron 1 of the *Ocstamp* gene prevented expression of any detectable OC-STAMP mRNA and produced animals with only mononuclear osteoclasts. This is consistent with the gene structure of *Ocstamp*, in which only the first 14 amino acids are encoded by exon 1, the bulk of the protein (amino acids 15–348 of 498) being encoded by exon 2, and the remainder by exon 3. Presumably, the IRES, the LacZ and PGK cDNAs, and the strong polyadenylation site of the gene trap inserted into intron 1 successfully blocked any transcription into exon 2, resulting in a *bona fide* loss-of-function allele. The flippase and Cre recombination sites are intact in this mouse. This leaves open the possibility of using Cre to excise the entire Neo selection cassette along with exon 2, or to proceed in 2 steps, first using flippase to delete the Neo cassette, then using an appropriate Cre driver to specifically delete exon 2, resulting in an OC-STAMP loss-of-function in cells or tissues of interest.

The previous report of OC-STAMP loss-of-function showed that, while knockout mouse osteoclasts had profound defects in resorption *in vitro*, paradoxically, the mice lacked a skeletal phenotype as measured by micro-CT [14]. This was particularly unexpected since DC-STAMP KO mice showed similar defects in resorption and had increased trabecular bone volume [13]. The 2012 report, however, did not include information on animal ages, number analyzed, or genders, so we undertook to investigate mice derived from ES cells carrying the OC-STAMP gene trap. Our results confirmed both aspects of that report, with the homozygous KO cells showing a 3.5-fold decrease in resorption capacity on HA plates, but with skeletal parameters examined by radiograph, micro-CT, or pQCT showing no significant differences between WT and OCSt-KO mice. The loss of resorbing capacity in vitro and fusion in vivo and *in vitro* was not reflected in alterations of expression of various other osteoclast genes, nor in significant changes in bone resorption or formation markers in serum. In particular, mRNA levels of the master osteoclast transcription factor, NFATc1, were not different between WT, OCSt-KO, or OCSt-KO osteoclasts rescued by viral transduction with OC-STAMP. The loss of fusion capacity of OCSt-KO osteoclasts therefore did not cause any apparent compensatory increases of other osteoclast effector genes. It is noteworthy that OCSt-KO osteoclasts are still capable of resorbing bone, but less efficiently.

We report here a number of skeletal parameters comparing OCSt-KO and WT mice and cells derived from them. All skeletal measurements, including radiographs, pQCT, and micro-CT, did not differ significantly, nor did quantitative measurements of the total number of TRAP-positive sites in bone sections. The total TRAP-positive area and femur metaphysis, however, was lower in the knockout mice, as was the mean area per site. Given that bone metabolic markers were not different between genotypes, indicative of normal bone formation and resorption, a reasonable inference is that the smaller osteoclasts are able to compensate for their reduced size by increased activity per cell to maintain skeletal mass and form.

The STAMPs are to date the only cell-type-specific factors shown to be required for pre-osteoclast and foreign body giant cell fusion, making their mechanism of action of particular interest. A number of other proteins with important roles in osteoclast fusion have been described, but their expression and activities are not restricted to pre-osteoclasts or giant cells. CD47 and its interacting receptor, SIRPα, have been shown to be important in macrophage/osteoclast fusion [26–28], but they are very widely expressed. Syncytin1, a retroviral fusion protein captured in the primate lineage some 24 million years ago, has been shown to be important in cell-cell fusion in human placental trophoblasts as well as human osteoclasts [7,29]. Syncytin1 is the only factor so far identified that takes part in the actual fusion of the lipid bilayers on adjoining cells. Its absence from other mammalian orders, however, means it cannot be a universal mediator of osteoclast membrane fusion. The gap junctional complex component, connexin 43, has also been shown to be important in osteoclast fusion, including producing pores that connect the cytoplasm of fusing cells. Connexin 43, however, is also widely expressed, most notably in cardiac myocytes, where its connections help to regulate synchronous contraction [30]. Interestingly, Søe and co-workers observed heterogeneity among populations of fusing OCs based on number(s) of nuclei, expression and localization of markers, and specific inhibitors [29]. They showed the DC-STAMP and CD47 were markers for fusing cells with low numbers of nuclei, i.e., relatively immature osteoclasts, whereas connexin 43 was a marker for more mature osteoclasts. They also found asymmetries in fusing cell mobility (low mobility, high number of nuclei, and vice versa) and reciprocity of adjoining structures: cups apposed to protrusions [29]. Recently, dynamin and clathrin-mediated endocytosis were shown to be necessary for the fusion of both pre-osteoclasts and myoblasts [10]. Those factors/processes, however, are present in essentially all cell types. How they are recruited and deployed during osteoclast fusion remains to be determined. It is conceivable, for example, that OC-STAMP and/or DC-STAMP are playing a role(s) in recruitment of dynamin to the actin-rich plasma membrane protrusions seen during fusion.

A critical role for plasma membrane protrusions/podosomes in pre-osteoclast fusion was recently demonstrated in studies of the intracellular adapter protein, Tks5 [9]. That report showed that Tks5 was induced during osteoclast differentiation, that it was phosphorylated by Src, and that it was downstream of PI3K. Without Tks5, both the formation of the actin belt that characterizes circumferential podosomes of osteoclasts and the fusion of pre-osteoclasts were inhibited. Interestingly, that report also showed that Tks5 was promoting the formation of plasma membrane protrusions which were enriched in PtdIns(3,4)P_2_ or PtdIns(3,4,5)P_3_ and which appeared to be generated from either podosome-like or filopodium-like protrusions of RAW264.7 cells during differentiation. Such filopodia are also called invadopodia in invasive tumor cells, and co-cultures of pre-osteoclasts with the invasive melanoma cell line B16F0 resulted in fused hybrid cells composed of both cell types. Whether DC- or OC-STAMP are involved in Tks5 activity has yet to be determined.

Knockout of either OC- or DC-STAMP blocks fusion at the one-cell stage. Considered in the context of the differentiation-stage-heterogeneity model described above, they are likely to be playing important roles in the very earliest steps in pre-OC fusion. This is consistent with DC-STAMP high and low cell populations previously described [31] in the sense that levels of the STAMPs may decrease as fusion progresses, as part of the heterogeneous fusion process; but that remains to be determined.

We investigated the localization and topology of OC-STAMP for several reasons. First, topological orientation of transmembrane proteins is critical to their functional interactions inside and outside the cell. Second, different topology prediction algorithms gave slightly different results for OC-STAMP [15]. Finally, in the future, OC-STAMP could conceivably represent a diagnostic or therapeutic target, and knowledge of its orientation could inform that development. We combined an approach using N- and C-terminal tags with cell permeabilization along with glycosylation studies.

The conservation of OC-STAMP during the evolution of land-based vertebrates is striking (**Table 1**), as is the lack of strong homologs in fish (not shown). Perhaps even more striking is the apparently absolute conservation of the glycosylation site at N162 in the mouse sequence. CLUSTAL alignments revealed that it is conserved in representatives of all orders and families of mammals for which sequence data are available, including ungulates, primates, rodents, and marsupials. It is also conserved in the classes *Aves*, *Reptilia*, and *Amphibia*. Whether OC-STAMP is regulating bone resorption in those classes in a manner analogous to mammals is not known, Given the conservation of the glycosylation motif, it was anticipated that loss of that site would compromise fusion. That was not the case in differentiation experiments *in vitro*; however, the low level of the non-glycosylated form of OC-STAMP protein remaining in differentiating osteoclast cultures suggests that glycosylation may be playing an important role in stability or recycling to maintain of high levels of OC-STAMP. It is also possible that important functions of glycosylation beyond protein stability are not evident in the in vitro osteoclast differentiation assays reported here.

A major issue in pre-osteoclast fusion is how the leaflets of the lipid bilayer are able to split and re-seal during fusion. Given that membrane fusion and scission of membrane-bounded organelles are universal in eukaryotic cells, it seems likely that osteoclasts in terrestrial vertebrates have evolved a specialized adaptation of those processes to achieve cell-cell fusion. Whether, and to what extent, OC-STAMP and DC-STAMP may hijack conserved intracellular machinery to achieve cell-cell fusion in pre-osteoclasts is an important and open question.

## Supporting Information

S1 Fig Statistical analyses of micro-CT data from 2-week-old WT and OCSt-KO mice. (TIF)

S2 Fig Statistical analyses of micro-CT data from 6- week-old WT and OCSt-KO mice. (TIF)

S3 Fig Statistical analyses of pQCT data from 6-week-old WT and OCSt-KO mice. (TIF)

S1 Table Sequences of forward and reverse primers used in Q-PCR. (DOCX)

S2 Table Micro-CT data from 6-week-old animals (Excel file). (XLS)

S3 Table pQCT data from 2 and 6-week-old animals (Excel file). (XLS)

S4 Table Mean area of TRAP stained osteoclasts. BMMC from WT and OCSt-KO mice were transduced with lentiviral vectors. WT BMMC were transduced with GFP alone, and OCSt-KO BMMC were transduced with GFP alone or with WT OC-STAMP fused to GFP at its C-terminus (experiment 1). In experiment 2, WT BMMC were transduced with GFP alone, and OC-STAMP BMMC were transduced with WT OC-STAMP fused to GFP or with (N162D) OC-STAMP fused to GFP. Cells were cultured for 6 days under differentiation conditions and stained for TRAP. The areas of the first 20 (± 1) osteoclasts seen in the wells (3 nuclei and more) were measured using NIH Image J software. Each experiment was repeated 3 times for a total of 60± 2 cells. Statistical analysis by *t*-test (exp. 1) or ANOVA (exp. 2) was done using GraphPad Prism 6 software.
(DOCX)
