# Supplementary figures and images for: Studies of OC-STAMP in Osteoclast Fusion: A New Knockout Mouse Model, Rescue of Cell Fusion, and Transmembrane Topology
Source: PLoS One. 2015 Jun 4;10(6):e0128275. doi: 10.1371/journal.pone.0128275 (PMC4456411; doi:10.1371/journal.pone.0128275)

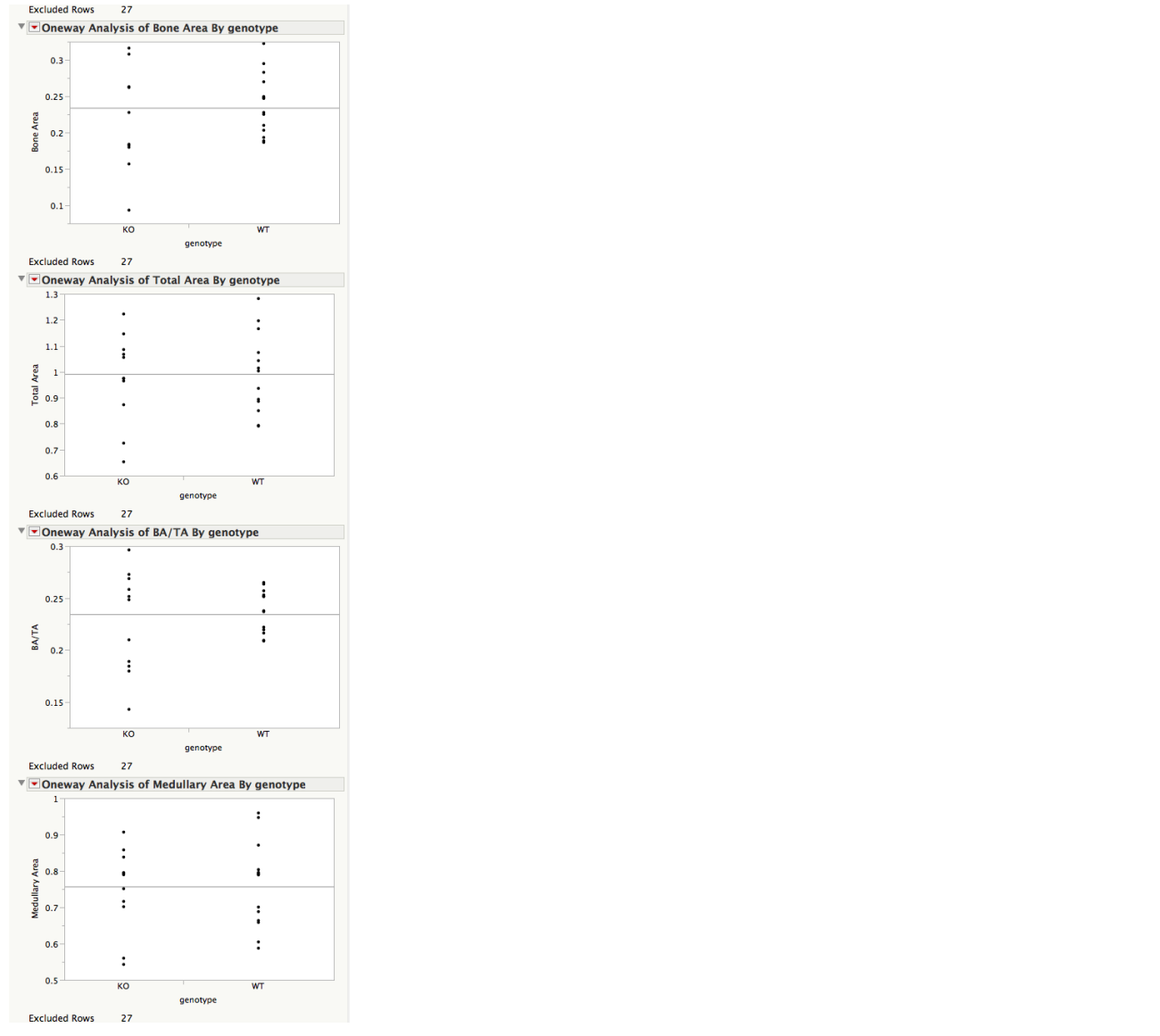

Supplement: S1 Fig — (TIF) [file pone.0128275.s001.tif]

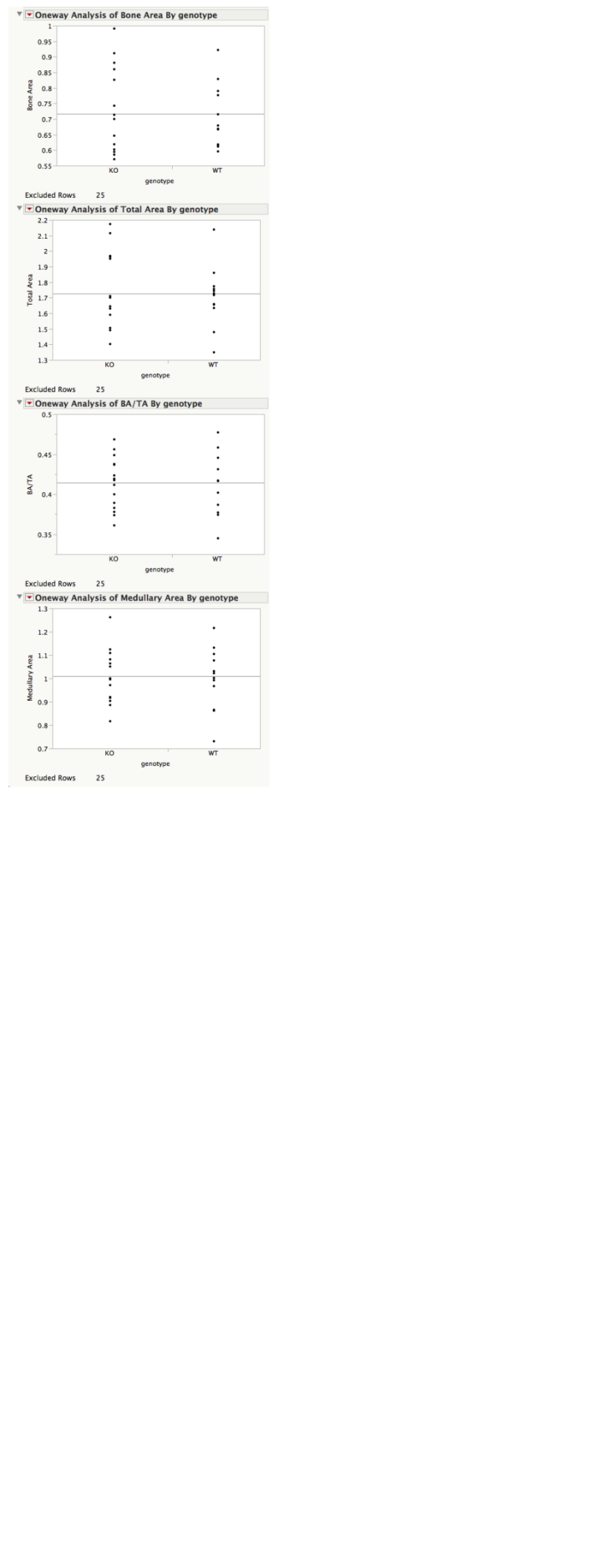

Supplement: S2 Fig — (TIF) [file pone.0128275.s002.tif]

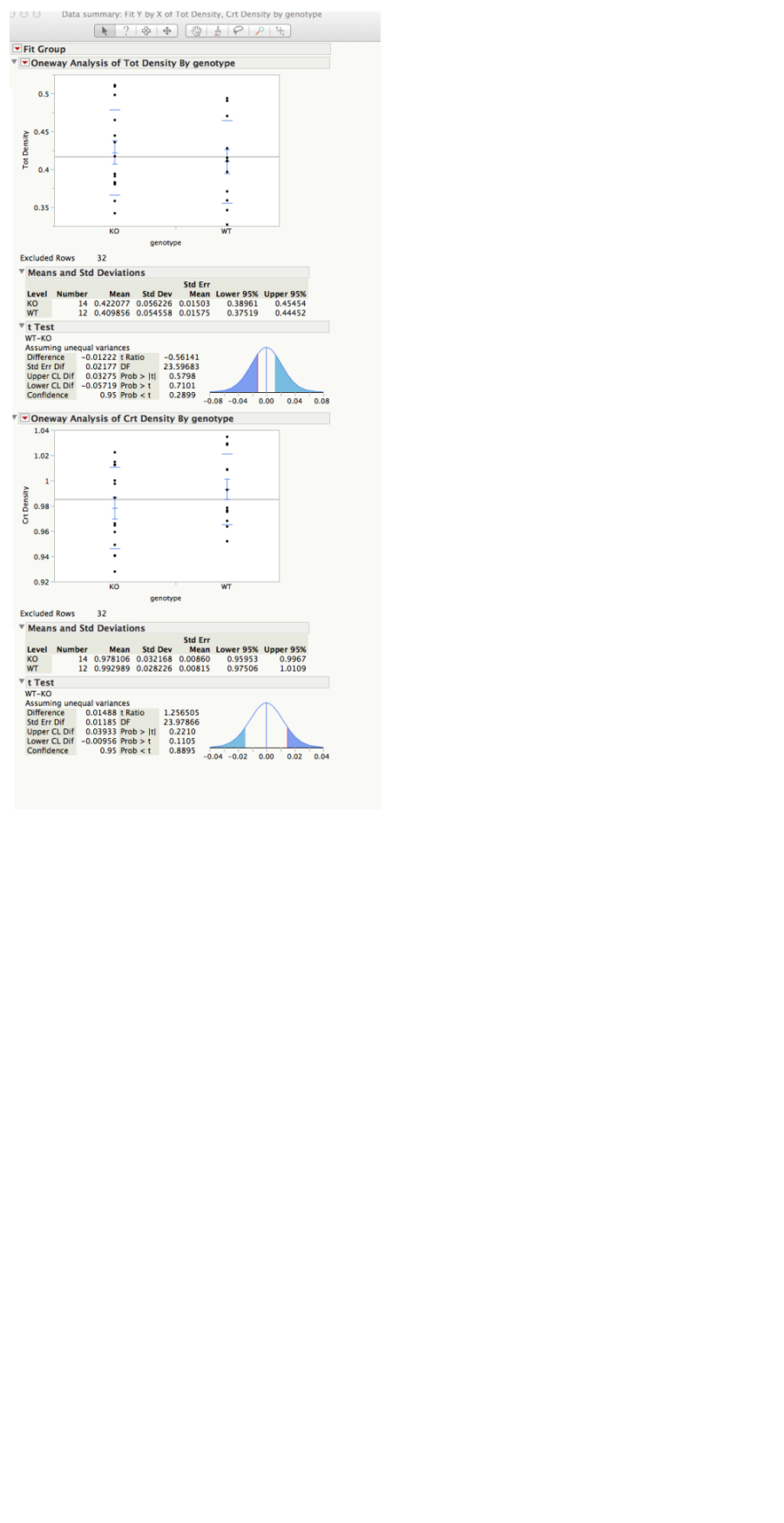

Supplement: S3 Fig — (TIF) [file pone.0128275.s003.tif]
