# Supplementary material for: Studies of OC-STAMP in Osteoclast Fusion: A New Knockout Mouse Model, Rescue of Cell Fusion, and Transmembrane Topology
Source: PLoS One. 2015 Jun 4;10(6):e0128275. doi: 10.1371/journal.pone.0128275 (PMC4456411; doi:10.1371/journal.pone.0128275)
Supplement: S1 Table — (DOCX) [file pone.0128275.s004.docx]

**S1 Table**

| **Gene** | **Primer sequence** |
| --- | --- |
| *Rplp0* (60S acidic ribosomal protein P0) | Forward 5’-TGTTTGACAACGGCAGCATTT-3’  Reverse 5’- CCGAGGCAACAGTTGGGTA -3’ |
| *Ca2* (Carbonic anhydrase II) | Forward 5’- CTCAGGGAGCCCATTACTGT -3’  Reverse 5’- TCCTCATTGAAGTTCAGCGT -3’ |
| *Ctsk* (Cathepsin K) | Forward 5’- GATGAAATCTCTCGGCGTTT -3’  Reverse 5’-CACTGGTCATGTCTCCCAAG -3’ |
| *Tm7sf4* (DC-STAMP) | Forward 5’- TGGAAGTTCACTTGAAACTACGTG -3’  Reverse 5’- CTCGGTTTCCCGTCAGCCTCTCTC -3’ |
| *Nfatc1* (nuclear factor of activated T-cells, cytoplasmic 1) | Forward 5’- CTCGAAAGACAGCACTGGAGCAT -3’  Reverse 5’- CGGCTGCCTTCCGTCTCATAG-3’ |
| *Ocstamp* (OC-STAMP) | Forward 5’- TTGCTCCTGTCCTACAGTGC -3’  Reverse 5’- GCCCTCAGTAACACAGCTCA-3’ |
| *Acp5* (Tartrate resistant acid phosphatase, TRAP) | Forward 5’-CCAGCGACAAGAGGTTCC-3’  Reverse 5’- AGAGACGTTGCCAAGGTAT-3’ |
