# Supplementary material for: Studies of OC-STAMP in Osteoclast Fusion: A New Knockout Mouse Model, Rescue of Cell Fusion, and Transmembrane Topology
Source: PLoS One. 2015 Jun 4;10(6):e0128275. doi: 10.1371/journal.pone.0128275 (PMC4456411; doi:10.1371/journal.pone.0128275)
Supplement: S4 Table — BMMC from WT and OCSt-KO mice were transduced with lentiviral vectors. WT BMMC were transduced with GFP alone, and OCSt-KO BMMC were transduced with GFP alone or with WT OC-STAMP fused to GFP at its C-terminus (experiment 1). In experiment 2, WT BMMC were transduced with GFP alone, and OC-STAMP BMMC were transduced with WT OC-STAMP fused to GFP or with (N162D) OC-STAMP fused to GFP. Cells were cultured for 6 days under differentiation conditions and stained for TRAP. The areas of the first 20 (± 1) osteoclasts seen in the wells (3 nuclei and more) were measured using NIH Image J software. Each experiment was repeated 3 times for a total of 60± 2 cells. Statistical analysis by t-test (exp. 1) or ANOVA (exp. 2) was done using GraphPad Prism 6 software. (DOCX) [file pone.0128275.s007.docx]

| **Exp.1.** Mean osteoclast area in WT and OCSt-KO BMMC Lentiviral Rescues | | | | |
| --- | --- | --- | --- | --- |
|  | **OC area (μm^2^)** | **SD** | **N** | ***P* value** |
| **WT** | 45,399 | 7,230 | 58 |  |
| **OC-STAMP:GFP Rescue** | 18,536 | 2,054 | 60 | *P*<0.0005 *vs*. WT |
| **Exp. 2.** Mean osteoclast area in WT and OCSt-KO BMMC Rescued with WT OC-STAMP or with OC-STAMP N162D. | | | | |
| **WT** | 48,315 | 5,090 | 60 |  |
| **OC-STAMP:GFP Rescue** | 14,655 | 1,599 | 61 | *P*<0.0001 *vs*. WT |
| **OC-STAMP(N162D):GFP Rescue** | 10,827 | 838 | 60 | *P*<0.0001 vs. WT;  *P>0.05 vs*. WT OC-STAMP:GFP Rescue |
